# Riemannian Metric Learning for Alignment of Spatial Multiomics

**DOI:** 10.64898/2025.12.09.693237

**Published:** 2025-12-11

**Authors:** Peter Halmos, Yufan Xia, Benjamin J. Raphael

**Affiliations:** 1 Department of Computer Science, Princeton University, 35 Olden St, Princeton, NJ 08544

## Abstract

Recent spatial technologies measure the transcriptome, epigenome, proteome, metabolome and other modalities from thousands of cells across a tissue. Most assays typically profile only one modality from a tissue slice, raising the question of how to align spatial data from heterogeneous feature spaces. While multiple approaches have been developed for multi-modal integration of single-cell datasets, few existing techniques perform spatial alignment across arbitrary modalities incorporating both spatial and feature information. We introduce Manifold Gromov-Wasserstein (MGW), a metric-learning framework that exploits the product structure of spatial multiomics to infer modality-specific Riemannian *pull-back* metrics with neural fields. MGW aligns Riemannian distances induced by these metrics via Gromov-Wasserstein optimal transport, yielding a hyperparameter-free cost across arbitrary modalities sharing a spatial base. The formulation enjoys theoretical invariances – including orthogonal transformations of the spatial and feature domains as well as global feature scalings. We demonstrate the advantages of MGW on multiple alignment tasks, including Stereo-Seq spatiotemporal transcriptomics of mouse embryo, Xenium and Visium spatial transcriptomics of colorectal cancer, and spatial metabolomics-transcriptomics from human striatum and kidney cancer. MGW recovers biologically meaningful correspondences and spatially coherent tissue structures, outperforming existing OT- and non-OT-based multi-modal baselines.

## 1 Introduction

Spatially-resolved transcriptomic (SRT) technologies (48; 42; 36) are a seminal advance towards a comprehensive understanding of spatial biology (2; 24), but they measure only a single of many biological modalities which define the state of a cell. Beyond transcription, a cell state is defined by modalities such as its metabolome, epigenome, and proteome. More recent spatial multiomics technologies combine measurements of the transcriptome with profiling of the epigenome (60) in spatial ATAC-Seq and spatial CUT & Tag-RNA-Seq, profiling of spatial metabolomics and histology (56), profiling of open chromatin and T-cell receptor sequences (43), and profiling of protein markers (4). However, most technologies only measure a single modality for a given slice. While modern computational techniques have offered great promise for understanding biology from a functional lens – from understanding cell-differentiation dynamics (46; 47; 45; 16) to spatial heterogeneity (51; 21; 13; 58; 6) and cellular niches (19; 14) – extending such questions to the spatial multiomic universe requires *alignment* across differing modalities.

Multiple works have tackled the problem of aligning spatial transcriptomics data (26; 7; 30; 59; 17). Techniques for spatial alignment have proven highly effective, yet universally face the difficult problem of how to weight spatial similarity against feature similarity. Some methods such as STAlign (7) avoid the question by relying on spatial information alone. Another popular approach called *fused Gromov-Wasserstein* (FGW) optimal transport (54), introduces a trade-off hyperparameter α between spatial and feature information. FGW was first introduced to spatial transcriptomics by PASTE (59) and adapted by many following works to account for partial alignment (30), unbalanced and semi-relaxed alignment (26; 17), and learning cell-fate landscapes (23). However, its hyperparameter is often difficult to tune and the framework is not extendable to multiple modalities.

Alignment of single-cell datasets from different modalities is also well-studied. Multimodal alignment presents a unique challenge, as these spaces may have differing dimensions, incomparable scales and geometries, and no common basis of coordinates on which points can be compared. However, aligning across heterogenous spaces is the most general and powerful form of alignment: it subsumes any notion of mode, from experimental batch to time point to technology. Works such as SCOT (12; 11), SCOTv2 (10), and moscot (26) align single-cell datasets across modalities using Gromov-Wasserstein optimal transport, matching the modality-spaces based on their pairwise distance structures. In contrast, numerous non-OT approaches – based on variational autoencoders, nearest-neighbor anchoring, or matrix-factorization (18; 49; 57; 29) – perform multiomic integration by learning a shared latent representation, but typically do not return explicit cross-dataset alignments. A universal limitation of existing OT-based multimodal alignment methods is that they do not leverage spatial information and do not *learn* a cost function. Instead, they rely on Euclidean or graph distances computed symmetrically within each modality on the raw features.

One approach to spatial multi-modal integration is to learn joint embeddings of spatial multiomics via graph representations or autoencoders, as in MultiGate (34) and SpatialMeta (53) but these methods presume *pre-aligned* cells. Even when an alignment module is included for spatial multiomics (e.g. using STAlign (7) in (53)), registration operates on *coordinates*, not on the feature-space geometry. Another recent and parallel line of work has investigated learning of the *Riemannian metric* of the data-manifold (1; 44), with applications to learning the latent manifold with autoencoders (50; 38). However, no existing techniques have bridged learning modality-specific metrics for the underlying spatial-molecular manifold with alignment across heterogeneous spaces.

### Contributions.

We introduce *Manifold Gromov-Wasserstein* (MGW), an algorithm to align spatial multiomics datasets across arbitrary modalities. The key insight of MGW is to learn a cost whose respective mapping across modality spaces preserves the *intrinsic manifold structure* of each space. MGW is based on the observation that a spatial ’omics dataset can be viewed as a function from physical (Euclidean) space $E\subset R^{2}$ or $R^{3}$ to a high-dimensional feature, or modality, space ℳ. As each modality shares the same base space E, MGW utilizes a standard approach in Riemannian geometry for studying different manifolds sharing a common base space: the *Riemannian pull-back metric*, which pulls the geometry of the modality spaces back onto the common Euclidean space. To compute the pull-back metric, we represent each spatial-omic dataset with a *neural field*: an implicit map from physical space into the respective modality space. Finally, MGW computes an alignment using Gromov-Wasserstein Optimal Transport on the Riemannian distances in each modality. Manifold Gromov-Wasserstein offers a geometrically-unified technique for spatial multimodal alignment and is free of hyperparameters for weighting between spaces.

We show that MGW is a reasonable measure of "distance" between spatial-feature manifolds, with invariance to rigid-body transformations of the spatial coordinates and invariance to orthogonal transformations, offsets, and homogeneous scalings of the features. Additionally, MGW recovers GW on spatial coordinates when the fields are identity maps. We demonstrate that MGW outperforms existing spatial alignment methods across four different spatial-omics alignment tasks spanning five measurement technologies. MGW bridges neural representation and feature-learning with optimal transport, offering a natural way to *learn* costs for optimal transport to align across different modalities.

## 2 Methods

A spatial-omics dataset $𝒟={\left(s^{i},X^{i}\right)}_{i=1}^{n}$ is a set of pairs of spatial locations $s^{i}$ and associated features $X^{i}$. For spatial multiomics alignment one has a common Euclidean base space E (e.g. $E=R^{2}$, $R^{3}$), and a pair of spaces $ℳ\subset R^{d}$, $𝒩\subset R^{p}$ representing the space for each measured modality (e.g. ℳ transcription, and 𝒩 metabolomics). We begin by introducing the general problem of aligning such multimodal data with a common Euclidean base space.

### **Problem 1** (Multimodal Alignment with Common Base Space.).

*Given spatial datasets* ${\left(s^{i},X^{i}\right)}_{i=1}^{n}\subset E\times ℳ$ *and* ${\left(t^{j},Z^{j}\right)}_{j=1}^{m}\subset E\times 𝒩$ *with modality spaces* ℳ *and* 𝒩 *over a common Euclidean base* E, *and given a cross-space cost* $C_{M\times N}\;:(E\times ℳ)\times (E\times 𝒩)\to R$,^1^ *find a coupling* $\pi^{*}$ *between the modalities* ^2^, *which minimizes the expected cost*

(1) $$\underset{\pi }{\text{min}}\;E_{(i,j)∼\pi }C_{M\times N}\left(\left(s^{i},X^{i}),(t^{j},Z^{j}\right)\right).$$

For fixed cost $C_{M\times N}(\cdot ,\cdot )$, Problem 1 is well-defined and the best alignment can be found using optimal transport (OT) (See (10) and (11)). The true challenge, then, is in determining or learning the most appropriate notion of cost (39). As different modality spaces exhibit incomparable dimensions, scalings, and geometries, identifying such a cost is difficult. A commonly used cost in spatial transcriptomics alignment is the Fused Gromov-Wasserstein (FGW) cost, which is defined to be a convex combination of a cost on feature-distances evaluated *across* datasets and a cost on spatial distances evaluated *within* a dataset (54; 59; 30; 26). This, however, poses existential limitations for Problem 1: FGW requires distances across the two modalities while its underlying cost functions are within one space, e.g. $\ell_{2}$ distances.

The key principle behind MGW is to not pre-define a cost, but to instead *learn* a cost which preserves the *intrinsic manifold structure* of each modality space. This is not only to map across heterogeneous spaces, but also to preserve non-linear manifold geometry. Even when two datasets exist in spaces which are not directly comparable, the two modalities may exhibit a common geometry when viewed relatively within each space. To define such a cost, we make two key assumptions on our data:

(A1) The modality spaces are *Riemannian manifolds* (ℳ, g) and (𝒩, h) equipped with associated Riemannian metrics to measure distances.
(A2) A pair of smooth mappings exist from the base Euclidean space into the respective modality spaces. We denote these by φ:E→ℳ and ψ:E→𝒩.

We note (A1) automatically holds for the base Euclidean space (E, $g^{E}$) with metric being the identity $g^{E}=\mathrm{id}$. Given (A1) and (A2), the core question of our work then becomes the following:

Can we learn a cost between arbitrary modalities measured over Euclidean space which can preserve the intrinsic geometry of the underlying data manifolds?

#### 2.1 Manifold-Pullback and Pullback Metric

In this section, we describe how MGW learns modality-specific notions of distance and pulls them back to the underlying Euclidean space. To make this precise, we first recall a few preliminaries from Riemannian geometry. Given a smooth manifold M, a *Riemannian metric* or *metric tensor* is assigns to each point p∈M a symmetric, positive-definite bilinear form $g_{p}\;:T_{p}M\times T_{p}M\to R$ on the tangent space $T_{p}M$ such that the coefficients $g_{ij}(p)$ vary smoothly in every local coordinate chart. The metric acts as an inner product on tangent vectors in $T_{p}M$ and defines a local notion of length and angle. When $M=R^{d}$, the canonical choice g(X,Y)≔〈X,Y〉 yields the standard Euclidean geometry. The pair (M, g) is called a Riemannian manifold (see, e.g., (27; 28) for standard references).

**Figure 1:**
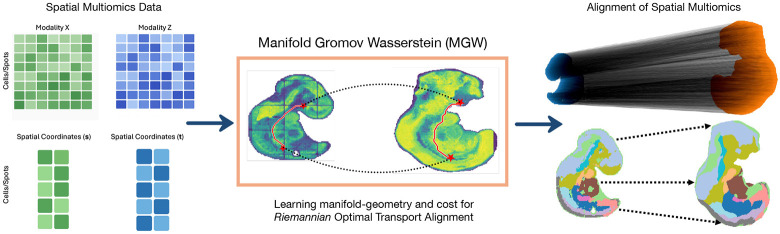
Overview of Manifold Gromov-Wasserstein (MGW). (Left) Two spatial multiomic datasets are input including both features for each modality and corresponding spatial coordinates. (Middle) MGW learns the intrinsic Riemannian geometry of each dataset with neural fields and aligns Riemannian distances. (Right) The alignment offers a non-linear mapping between cells measured in arbitrary modalities while preserving the intrinsic geometry of each dataset.

To extend these objects to spatial-multiomics, we need to learn the geometry of the features over physical space. We note that a spatial dataset $𝒟={\left(s^{i},X^{i}\right)}_{i=1}^{n}$ can be viewed as n samples ${\left(s^{i},\varphi (s^{i})=X^{i}\right)}_{i=1}^{n}$ of tissue coordinates and a function φ:E→ℳ mapping the tissue coordinates (Euclidean space) into the modality space ℳ. We represent this function for each modality using an *implicit neural field* or *implicit neural representation* φ:E→ℳ, ψ:E→𝒩. Fields offer a different perspective on spatial multiomics: rather than representing the data as a finite grid or matrix of spatial and feature coordinates (Figure 1), one views the data *implicitly* as a continuous field of features over physical space. Spatial multiomics data are well-suited to field-based parametrizations: gene expression or other molecular modalities vary smoothly and continuously across tissue (6), and exhibit non-linear *isodepth* coordinates along which expression changes with a differentiable gradient (6; 31). For MGW, the key value of the field-based representation is in capturing the spatial differential structure underlying modality variation over space. With access to spatial derivatives $\partial_{x}^{k}\varphi_{\theta }$, one can access essential geometric objects – gradients, curvature, and induced metric tensors.

Following (6), we learn these field representations by minimizing the mean-squared loss

(2) $$\theta =\underset{\theta }{\text{arg min}}\;\frac{1}{2n}\sum_{i=1}^{n}{\left\|\varphi_{\theta }(s^{i})-X^{i}\right\|}_{2}^{2},\;\;\eta =\underset{\eta }{\text{arg min}}\frac{1}{2m}\sum_{j=1}^{m}{\left\|\psi_{\eta }(t^{j})-Z^{j}\right\|}_{2}^{2}$$

And taking $\psi ≔\psi_{\eta }$, $\varphi ≔\varphi_{\theta }$ to be the implicit representations of each omics slice defined as a field defined over Euclidean space. For our architecture, we restrict to $C^{\infty }$ activations; in particular, we use the SiLU function (41; 35). This ensures that $\psi_{\eta }$, $\varphi_{\theta }$ have smooth Jacobians and represent smooth submanifolds, when viewed as local parametrizations mapping from Euclidean space E into the respective modalities. Many Jacobians in neural representations of omics data play key roles: gene-over-gene Jacobians characterize temporal gene-regulatory networks (47) and gene-over-space or modality-over-space Jacobians (Definition 1) characterize the gradients and curvature of a modality over physical space (6; 31). As the modality-over-space Jacobian is central to this work, we recall it below.

### Definition 1 (Space-Modality Jacobian.).

*Let* φ:E→M *denote a field mapping spatial coordinates* $s\in E\subset R^{d_{E}}$ *to a feature-space* $M\subset R^{d}$. *At each point* s, *the* space-modality *Jacobian is the differential*

(3) $$J_{\varphi }(s)={\left[\frac{\partial }{\partial s_{j}}\varphi^{i}(s)\right]}_{ij}$$

(3) encodes how spatial displacements change modality (feature) abundances. As s varies over E, $J_{\varphi }(\cdot )\;:E\to L(TE,T_{\varphi }M)$ defines a smooth *Jacobian tensor field* of order (1, 1) on E. In other words, each point s in E maps to a Jacobian encoding the manner in which spatial displacements change features at that point. Now, given the Jacobian (3), we define the *pullback metric* of the fields φ, ψ onto the Euclidean base E.

### Definition 2 (Modality Pullback Metric).

Given a field φ with Jacobian field $J_{\varphi }(\cdot )\;:E\to L(TE,T_{\varphi }M)$, at a point x the Pullback-Metric induced by φ for modality M is

(4) $$g^{M}(x)=J_{\varphi }{\left(x\right)}^{T}J_{\varphi }(x)$$

*A symmetric, positive-definite* (0, 2) *tensor on the tangent space* $T_{x}E$. *The associated inner product is given by*

(5) $$g^{M}(x)(u,v)≔\langle J_{\varphi }(x)u,J_{\varphi }(x)v\rangle$$

*Observe that* $g^{M}(\cdot )\;:E\to S^{2}(T^{*}E)$ *defines a smooth* (0, 2) *tensor-field over* E.

Given our pair of smooth neural fields mapping from Euclidean space into the respective modality spaces, each mapping implicitly defines a Riemannian pull-back metric on the base space through the Jacobians $J_{\varphi }$ and $J_{\psi }$. The Riemannian pull-back metric is a classical approach in differential geometry for comparing manifolds in different spaces which may be expressed as maps from a common space.

While we offer a more formal description of this pull-back metric in Section A.1, this metric has a simple interpretation. Intuitively, $g^{M}(x)$ (4) encodes the local anisotropy of modality/feature variation along different spatial directions in the modality M. A pair of points is deemed close if small spatial perturbations induce similar changes in the modality field. Thus, it "pulls" modality-specific structure back on the common Euclidean space. By mapping from Euclidean space into the modality spaces we are allowed to work in reference to the space common to two spatial multiomics datasets and compare distances in this common space.

For any pair of points p, q∈M, recall the *Riemannian (geodesic) distance* between them is given by the minimal length of a smooth curve γ:[0,1]→M connecting them as measured in the metric

(6) d(p,q)≔inffγ∈C∞[0,1]{∣∫01g(γ(s))(γ.(s),γ.(s))ds∣γ(0)=p,γ(1)=q}

This coincides with the Euclidean distance when M is flat, and captures shortest paths in space curved according to the metric g. Now, given the pair of pull-back metrics for the two modalities defined on the common Euclidean space E

$$g^{M}(p)=J_{\varphi }{\left(p\right)}^{T}J_{\varphi }(p),\;\;g^{N}(q)=J_{\psi }{\left(q\right)}^{T}J_{\psi }(q)$$

One may define Riemannian distances with respect to the pull-backs $g^{M}$ and $g^{N}$ for each modality (Definition 4). In particular, for each pair of spatial points s, $s^{\prime }\in E$ in our first dataset and t, $t^{\prime }\in E$ in our second, we compute geodesics $\gamma^{M}$, $\gamma^{N}$ in the modality-specific metrics $g^{M}$, $g^{N}$ and their Riemannian distance:

(7) dM(s,s′)≔inffγM∈C∞[0,1]{∣∫01gM(γ)(γ.(s),γ.(s))ds∣γ(0)=s,γ(1)=s′},

(8) $$\text{resp}.\;\;d^{N}(t,t^{\prime })$$

**Figure 2:**
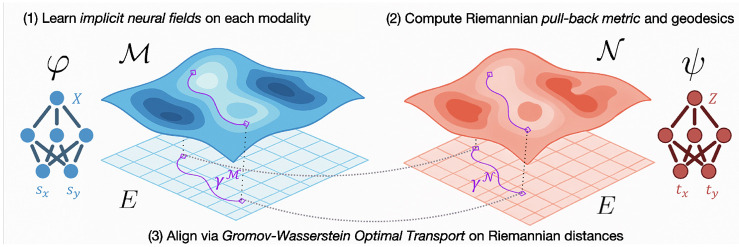
Manifold Gromov-Wasserstein (MGW). φ, ψ map from a Euclidean space E to distinct modality spaces ℳ and 𝒩. The maps φ, ψ define Riemannian pull-back metrics $g^{M}$ and $g^{N}$ through their Jacobians, from which one can compute geodesics $\gamma^{ℳ}$, $\gamma^{𝒩}$. Gromov-Wasserstein optimal transport yields an optimal alignment with respect to the Riemannian geodesic distances.

These paths $\gamma^{M}$ and $\gamma^{N}$ are highly intuitive (see, e.g. Figure 2), corresponding to a pull-back of the geodesics on each modality space onto E through the special requirement that φ(E)=ℳ and ψ(E)=𝒩. Following training of the neural fields φ, ψ, computation of $g^{M}$ and $g^{N}$ at each point relies evaluation of the Jacobian, which is easily accomplished with automatic differentiation (3). However, (7) requires computing continuous, shortest-path geodesic curves. To approximate these, we simply discretize these paths at the resolution of the underlying spatial coordinate grid. In particular, given these metrics the geodesics are computed by building a k-NN graph ${𝒢}_{M}$ on the spatial points $\{s^{i}\}\subset E$ with weights given by the local arc-length (infinitesimal distance unit) in the Riemannian metric between each neighboring pair of points. For edge (i, $i^{\prime }$) one may define $\Delta_{ii^{\prime }}=(s^{i}-s^{i^{\prime }})$ and compute the local, symmetrized arc-length as

$$w_{M}^{ii^{\prime }}=\frac{1}{2}\sqrt{\Delta_{ii^{\prime }}^{T}g_{i}^{M}\Delta_{ii^{\prime }}}+\frac{1}{2}\sqrt{\Delta_{ii^{\prime }}^{T}g_{i^{\prime }}^{M}\Delta_{ii^{\prime }}}.$$

One repeats this for the second modality space 𝒩 and constructs $w_{N}^{jj^{\prime }}$ with graph ${𝒢}_{N}$ on the spatial coordinates $\left\{t^{j}\right\}$ of the second dataset. Now, with both spatial coordinates (vertices) and arc-lengths in the metric (edge weights), the Riemannian geodesic distances (7) are easily computed with standard routines for all-pairs shortest paths (APSP). Graph-based approximation of Riemannian geodesics on a k-NN graph is standard in manifold-learning and geometric graph methods (52; 8), and scales well to typical spatial omics resolutions.

#### 2.2 Alignment with Manifold Gromov-Wasserstein

We begin by recalling the Wasserstein formulation of optimal transport (25). Let $\left\{x^{1},\cdots ,x^{n}\right\}$ and $\left\{y^{1},\cdots ,y^{m}\right\}$ be two datasets, and denote $\Delta_{k}$ to be the probability simplex of size k. In optimal transport, one encodes the two datasets as probability measures $\mu =\sum_{i}a_{i}\delta_{x^{i}}$ and $\nu =\sum_{j}b_{j}\delta_{y^{j}}$ with probability vectors $a\in \Delta_{n}$, $b\in \Delta_{m}$ representing the weight of each point (e.g. uniform). Then, define the set of couplings with marginals a and b to be

(9) $$\Pi_{a,b}≔\{P\in R_{+}^{n\times m}\;:P1_{m}=a,\;P^{T}1_{n}=b\}$$

Given a cost matrix of distances between the points $X^{i}$, $Y^{j}$, $C_{ij}≔c(X^{i},Y^{j})$, Wasserstein optimal transport (25) seeks a coupling P of minimal cost with respect to c

(10) $$W_{c}(\mu ,\nu )≔\underset{P\in \Pi_{a,b}}{\text{min}}{\langle C,P\rangle }_{F}.$$

Where ${\langle A,B}_{F}=\sum_{ij}A_{ij}B_{ij}$ denotes the Frobenius (matrix) inner product. While the Wasserstein formulation finds a coupling or alignment minimizing the distances between two points in a common space, often one seeks to compare $\{X^{1},\cdots ,X^{n}\}\subset 𝒳$ and $\{Y^{1},\cdots ,Y^{m}\}\subset 𝒴$ in distinct metric spaces 𝒳, 𝒴. *Gromov-Wasserstein* optimal transport (32; 33) addresses this by allowing two cost functions $c_{1}\;:𝒳\times 𝒳\to R_{+}$ and $c_{2}\;:𝒴\times 𝒴\to R_{+}$, and instead comparing the metric distortion under P

(11) $$\text{GW}(\mu ,\nu )≔\underset{P\in \Pi_{a,b}}{\text{min}}\sum_{ii^{\prime }jj^{\prime }}P_{ij}P_{i^{\prime }j^{\prime }}{\left(c_{1}(X^{i},X^{i^{\prime }})-c_{2}(Y^{j},Y^{j^{\prime }})\right)}^{2}.$$

The Fused Gromov-Wasserstein (FGW) objective function (55) is a convex combination of the Wasserstein 10 and Gromov-Wasserstein 11 objectives with hyperparameter α∈(0,1).

We define the *Manifold (Pull-Back) Gromov-Wasserstein Problem* to be the optimal coupling for (11) given the Riemannian pull-back distances 7 $d^{M}(\cdot ,\cdot )$ and $d^{N}(\cdot ,\cdot )$ of each modality onto the common base E.

### Problem 2 (Manifold Pull-Back GW Problem.).

Given pull-back distances (7) $d^{M}$, $d^{N}$ and distributions $a\in \Delta_{n}$ over ${\left(s^{i},X^{i}\right)}_{i=1}^{n}$ and $b\in \Delta_{m}$ over ${\left(t^{j},Z^{j}\right)}_{j=1}^{m}$, Problem 2 is the optimization

(12) $$P^{⋆}\in \underset{P\in \Pi (a,b)}{\text{arg min}}\sum_{ii^{\prime }}\sum_{jj^{\prime }}{\left(d^{M}{\left(s^{i},s^{i^{\prime }}\right)}^{2}-d^{N}{\left(t^{j},t^{j^{\prime }}\right)}^{2}\right)}^{2}P_{ij}P_{i^{\prime }j^{\prime }}$$

We highlight a few fundamental invariance and consistency properties of the MGW formulation.

(P1) (Consistency with Spatial GW) If the neural fields φ and ψ are identities; i.e. φ(s)=s and ψ(t)=t, then
$$\;g^{M}(x)=g^{N}(x)=I,\;d^{M}(x,x^{\prime })=d^{N}(x,x^{\prime })={\left\|x-x^{\prime }\right\|}_{2}.$$
  In the absence of information about the feature space, this offers an Occam’s Razor: (12) reduces exactly to the standard Gromov-Wasserstein problem on space E.
(P2) (Spatial Isometry Invariance, Proposition 1) Problem 2 is invariant to arbitrary translations $b\in R^{k}$, and orthogonal transformations $Q\in {𝒪}_{k}=\{Q\in R^{k\times k}\;:Q^{T}Q=QQ^{T}=I_{k}\}$, so that solving 2 on $s^{i}$ is equivalent to solving on
$${\tilde{s}}^{i}=Qs^{i}+b$$
(P3) (Feature Isometry and Scaling Invariance, Proposition 2) Let b, $b^{\prime }\in ℳ$, 𝒩 be any two constant vectors, let λ∈R:λ≠0 be a scaling, and $Q\in {𝒪}_{d}$, $U\in {𝒪}_{p}$ any two global orthogonal feature transformations. Then, the solution to Problem 2 is invariant to transformations of the feature space of the form
$$\hat{\varphi }(x)=\lambda \cdot Q\varphi (x)+b,\;\;\hat{\psi }(x)=\lambda \cdot U\psi (x)+b^{\prime }$$

Notably, MGW is invariant to the coordinate representation or parameterization of both the feature and spatial spaces. Even when trained on differing neural architectures or parametrizations of the feature and spatial space, these guarantees hold and ensure MGW behaves robustly. This is in contrast to coordinate-dependent formulations such as (53; 7).

Manifold-GW offers a geometric unification for spatial multiomics alignment: for distinct feature modalities and a shared base space, all comparisons factor through a single term on this base – without the need for trade-off hyperparameters like fused Gromov-Wasserstein. By using a mapping φ, ψ to pull the modalities back to a shared space, the distances $d^{M}(s^{i},s^{j})$ and $d^{N}(t^{i^{\prime }},t^{j^{\prime }})$ are over a common space E and are compatible for comparison in a sense that raw feature distances, $c_{M}(X^{i},X^{j})$ and $c_{N}(Z^{i},Z^{j})$ may not be. Thus, even when ℳ≠𝒩 are different spaces (e.g. representing distinct modalities), we are able to map directly between them by taking advantage of the product structure between the Euclidean space E and the pair ℳ, 𝒩.

We offer Algorithm 1 for MGW below, with an extended Algorithm S1 explicating all implementation details.

**Table:** 

Algorithm 1 (Manifold GW: Geodesic Alignment).
$$\begin{array}{cc} \; & \;(i)\;Learn\;neural\;fields.\;\text{Given samples}\;{\left(s^{i},X^{i}\right)}_{i=1}^{n}\subset E\times ℳ,{\left(t^{j},Z^{j}\right)}_{j=1}^{m}\subset E\times 𝒩,\;\text{learn}\;\varphi \equiv \varphi_{\theta }\;:E\to ℳ\\ \; & \;\;\;\text{and}\;\psi \equiv \psi_{\eta }\;:E\to 𝒩\;\text{which map spatial coordinates}\;E\;\text{to features in each modality:}\\ \; & \;\;\;\;\;\;\;\;\;\;\;\;\;\theta \leftarrow \text{arg}\underset{\theta }{\text{min}}\frac{1}{n}\sum_{i}{\left\|\varphi_{\theta }(s^{i})-X^{i}\right\|}_{2}^{2},\;\;\eta \leftarrow \text{arg}\underset{\eta }{\text{min}}\frac{1}{m}\sum_{j}{\left\|\psi_{\eta }(t^{j})-Z^{j}\right\|}_{2}^{2}\\ \; & \;(\text{ii})\;Compute\;pull\text{-}back\;metricks.\;\text{For each point}\;s^{i}\in E\;\text{and}\;t^{j}\in E,\;\text{evaluate}\\ \; & \;\;\;\;\;\;\;\;\;\;\;\;\;\;\;\;\;\;\;g^{M}(s^{i})=J_{\varphi }{\left(s^{i}\right)}^{T}J_{\varphi }(s^{i}),\;\;g^{N}(t^{j})=J_{\psi }{\left(t^{j}\right)}^{T}J_{\psi }(t^{j}),\\ \; & \;\;\;\text{where}\;J_{\varphi },J_{\psi }\;\text{are the space-modality Jacobians of neural fields}\;\varphi ,\psi .\\ \; & \;(\text{iii})\;Compute\;Riemannian\;geodesicdistances.\;\text{Using}\;g^{M},g^{N}\;\text{compute geodesics}\;\gamma^{M},\gamma^{N}\;\text{to obtain pairwise}\\ \; & \;\;\;\text{Riemannain distances}\;d^{M}(s^{i},s^{i^{\prime }})\;\text{and}\;d^{N}(t^{j},t^{j^{\prime }})\;\text{across the base manifold}\;E.\\ \; & \;(\text{iv})\;Solve\;Gromov-Wasserstein.\;\text{Given marginals}\;a\in \Delta_{n},b\in \Delta_{m},\;\text{solv}\\ \; & \;\;\;\;\;\;\;\;\;\;\;\;\;\;\;\;\;P^{⋆}=\text{arg}\underset{P\in \Pi (a,b)}{\text{min}}\sum_{ii^{\prime }jj^{\prime }}{\left(d^{M}{\left(s^{i},s^{i^{\prime }}\right)}^{2}-d^{N}{\left(t^{j},t^{j^{\prime }}\right)}^{2}\right)}^{2}P_{ij}P_{i^{\prime }j^{\prime }}.\\ \; & \;(v)\;Output\;the\;optimal\;coupling\;P^{*}. \end{array}$$

#### 2.3 On Identifying the Alignment-Informative Feature Set

Observe that in 2 one assumes that the two spaces ℳ and 𝒩 exhibit joint structure. Yet the feature spaces of distinct modalities may not fully align or exhibit meaningful joint structure. In particular, there may be "marginal" sub-spaces within a modality which may be entirely independent of any features in the other modality, and there may be "joint" sub-spaces which exhibit high-correlation between the modalities. Thus, a natural pre-processing is to filter for such features $\bar{ℳ}\subset ℳ$ and $\bar{𝒩}\subset 𝒩$ so that $\bar{ℳ}=\varphi (E)$ and $\bar{𝒩}=\psi (E)$ have high mutual information across s∈E. In Section C.6 we outline a pre-processing step to accomplish this by using CCA (*canonical correlation analysis* (20)) to filter for the subset of features across modalities with joint-structure prior to inferring the Riemannian metric with MGW.

## 3 Results

### 3.1 Spatiotemporal Transcriptomics of Mouse-Embryo

As an initial baseline and ablation of MGW, we benchmark on unimodal transcriptomics-transcriptomics data from consecutive time-points E9.5-10.5, E10.5-11.5, E11.5-12.5, and E12.5-13.5 of the mouse embryo using the Stereo-Seq platform (5). To mimic a multi-modal alignment and to isolate the impact of the proposed MGW cost, we focus the evaluation against standard Gromov-Wasserstein (GW) baselines: GW on Euclidean spatial distances, GW on Euclidean feature distances, unbalanced GW on feature distances with moscot TranslationProblem (26), and unbalanced GW on raw (Euclidean) feature geodesic distances with SCOTv2 (10).

**Figure 3:**
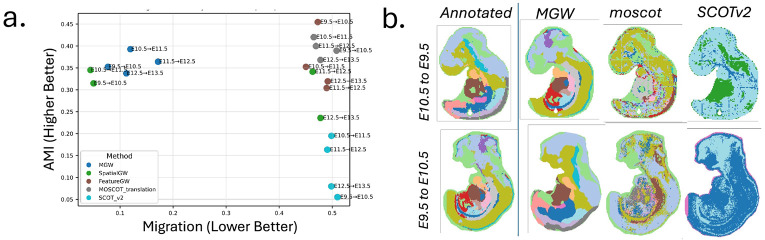
Comparison of Gromov-Wasserstein formulations on Stereo-Seq mouse-embryo timepairs E9.5-13.5 (5). (**a**) Comparison of alignments of pairs E9.5-10.5, E10.5-11.5, E11.5-12.5, and E12.5-13.5 in terms of migration distance and adjusted mutual information (AMI) with respect to published cell types. (**b**) Visualization of the most-likely cell-type (50% confidence threshold) predicted in the forward time direction (E9.5→E10.5) and backward time direction (E10.5→E9.5) transferred through each alignment.

We assess alignments along two axes of quality: a metric of spatial realism, and a metric of feature alignment (Figure 3a). For the spatial accuracy of the alignment, we compute the migration metric (59; 30; 17): the average displacement of a cell when mapped into the coordinate frame of the target slice. For interpretability, we report the migration as percentage of the maximal extent of the slide. For the biological accuracy of the mapping, we compute the adjusted mutual information (AMI) of the predicted cell-types under the coupling against the ground-truth cell-type annotations present in (5).

We find that MGW achieves the best balance between spatial realism and feature alignment (Figure 3a). MGW has expected cell-migration of 7.7, 12.0, 17.2, and 11.2 % of the slide compared to GW on spatial distances alone: 5.0%, 4.4%, 46.3%, and 47.8%. Moreover, MGW achieves higher AMI values than spatial GW for all pairs: 0.353, 0.393, 0.364, 0.338 versus 0.315, 0.345, 0.341, 0.236. Notably, for the latter time pairs (E11.5-12.5 and E12.5-13.5) MGW achieves even lower migration distances than spatial-only GW – in these cases, the non-convex spatial GW optimization can converge to a global flip relative to the ground-truth due to nearly spatially symmetric coordinates (Figure S10). By learning the metric for each modality, MGW can break such symmetries. Relative to performing GW on features alone, which yields unrealistically large migrations of 47.3, 45.0, 48.9, and 49.2 % of the slide, MGW achieves much lower migration while maintaining comparable AMI values (0.353, 0.393, 0.364, 0.338 versus 0.455, 0.352, 0.304, 0.319). Likewise, compared to moscot, which produces migration distances of 50.8%, 46.5%, 46.9%, and 47.8% with AMI scores of 0.389, 0.420, 0.400, and 0.368, and to SCOTv2, which exhibits migrations of 51.0%, 49.9%, 49.1%, and 49.8% with markedly lower AMI (0.055, 0.195, 0.163, and 0.080), MGW attains minimal spatial displacement while maintaining high biological coherence, as shown in Figure 3b which illustrates the predicted cell-types at ≥ 50% confidence. These results highlight the importance of spatial information to correctly align spatially structured tissues and underscore the value of aligning the intrinsic manifold as opposed to raw expression or spatial coordinates.

### 3.2 Visium and Xenium Alignment of Human Colorectal Cancer.

We perform an alignment of two multi-modal sections of a 10x Genomics colorectal cancer (CRC) dataset from the same donor (Sample P2 CRC) (37). This dataset includes a Visium CytAssist v2 section and a Xenium spatial transcriptomics section profiling the same tissue. While both datasets measure gene expression, they represent distinct measurement types: in Visium one performs sequencing of the full transcriptome (18k genes), while Xenium performs FISH imaging on a smaller panel of 422 genes. We benchmark 8 techniques on this dataset: MGW, Spatial GW, Feature GW, moscot (26), SCOT (12), SCOTv2 (10), PASTE2 (30), and POT (15). We measure alignment quality through (1) mean expression cosine similarity between common Xenium-Visium genes, and (2) the spatial migration distance between aligned spots. Strong alignments maintain high cosine similarity in aligned cells, while also minimizing spatial distortion and crossings in the alignment.

**Figure 4:**
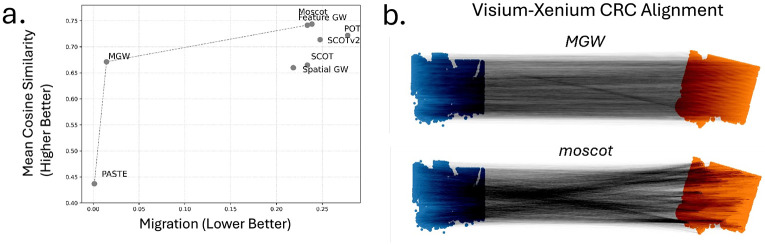
Comparison on Visium and Xenium datasets of colorectal cancer (37). (**a**) Cosine similarity and normalized migration of alignments on CRC Visium-Xenium alignment and (**b**) Visualization of MGW and moscot TranslationProblem alignment.

We find that MGW achieves the best balance with high cosine similarity (mean 0.671 and median 0.689) and low migration distance (average 1.4% of the slide) (Figure 4, Table 4). Most of the other techniques – including Feature GW, moscot, SCOT, and SCOTv2– align based on feature-information alone and ignore the constraint of spatial context. As a result, they exhibit higher mean cosine scores 0.744/0.742/0.665/0.714, respectively, but also a highly unrealistic amount of spatial migration of 23.9 %/23.4%/23.4%/24.8%, respectively, of the spatial extent of the slide for the cell-cell mapping (Figure 4a). As PASTE2 is not a multi-modal alignment method, it only uses spatial information and achieves a more realistic quantity of migration of only 0.1% of the slide but also a low expression cosine score of 0.437 indicating poor alignment of the transcription. Thus, MGW is the only technique with cosine-similarity at the level of feature-only methods and spatial distortion at the level of spatial-only methods.

### 3.3 Metabolomics and Transcriptomics Alignment of Human Clear Cell Renal Cell Carcinoma (ccRCC)

We evaluated MGW on two tissue slices from clear-cell renal cell carcinoma (ccRCC), one assayed with a AFADESI-MSI spatial metabolomics and the other with 10x Genomics Visium spatial transcriptomics (dataset Y7_T from (22)). We benchmark on the task introduced in (53), which consists of: (1) aligning the multi-modal spatial metabolomic-transcriptomic datasets, (2) projecting the metabolomic profiles onto the transcriptomic coordinates through the alignment, and (3) computing joint-embeddings with the variational autoencoder (VAE) architecture of SpatialMeta (53). We compare MGW to multi-modal single-cell optimal-transport (OT) based techniques SCOT (11), SCOTv2 (10), and moscot Translation (26), and to the non-OT based spatial metabolomics-transcriptomics method SpatialMeta (53), which introduced an Alignment Module based on STAlign (7) applied to the spatial coordinates. We also include Spatial-only GW and Feature-only GW as baselines. We ensure the training and architecture of the VAE are identical for all methods and repeat the entire evaluation pipeline, including alignment and training, across random seeds to ensure consistency. A complete summary of all metrics is provided in Table 2, and further details on our evaluation procedure and implementation may be consulted in Section C.

**Figure 5:**
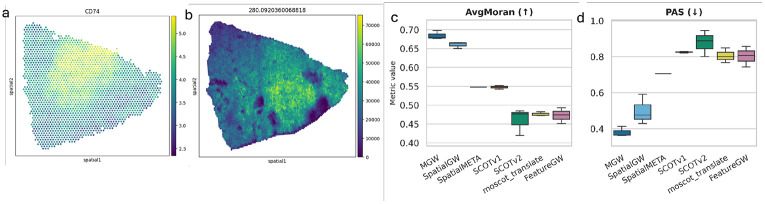
Cross-modal spatial transcriptomics and metabolomics alignment evaluation of MGW, spatial transcriptomics-metabolomics method SpatialMeta, and other OT baselines on the Y7_T slice from the ccRCC dataset (22). (**a**) The spatial distribution of gene *CD74* and (**b**) an associated metabolite with high Moran’s I. (**c**) Average Moran’s I on the two modalities and (**d**) PAS reported across three random seeds

We find MGW reported the highest average spatial coherence across all methods, as quantified by Moran’s I across both modalities (0.6845 ±0.0134) (Figure 5c). Moran’s I assesses whether the top spatial transcriptomics and spatial metabolomics marker features selected from the Leiden clusters exhibit spatially smooth, region-specific expression patterns. MGW substantially outperforms SpatialMeta (0.5478), which is specifically designed for spatial transcriptomic and metabolomic alignment.

MGW also outperforms the other methods on all three spatial continuity metrics used in (53) (Table 2). For example on PAS score, which quantifies how frequently a spot has neighbors belonging to a different cluster (see Section C), MGW achieved the lowest (best) value (Figure 5d), averaging 0.3797 across seeds, exceeding SpatialMeta (0.7063) as well as SCOT, SCOTv2 and moscot with scores of 0.8255, 0.8784, and 0.8058. In addition, the scores exceed those of Feature and Spatial GW (0.8029 and 0.4985, respectively). Techniques which aligned using spatial coordinates alone, such as SpatialMeta (which uses STAlign) and Spatial Gromov-Wasserstein, exhibited stronger performance on this task than ones relying on feature-information alone. This reflects the similarity of the aligned spatial grids (Figure 5a,b).

### 3.4 Spatial Metabolomics and Transcriptomics Alignment of Striatum in Human Brain

**Figure 6:**
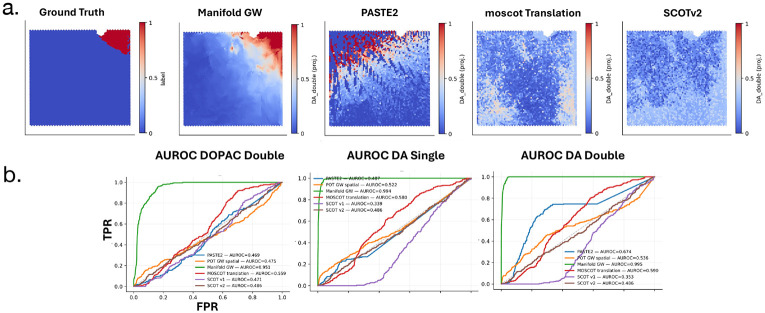
(**a**) (Left) Published (“Ground-truth”) dopamine labels on Visium slide (56). (Right) Normalized barycentric projection of doubly-derivatized dopamine onto Visium slide across methods. (**b**) AUROC curves for the overlap between the locations of Dopamine metabolites DA Single (singly-derivatized *m/z* = 421.19), DA double (doubly-derivatized *m/z* = 674.28), 3MT (dopamine-breakdown product 3-Methoxytyramine, *m/z* = 435.21), and DOPAC double (dopamine-breakdown product *m/z* = 698.24) and the annotated locations of dopamine-associated neurons on Visium spatial transcriptomics slide for each method.

Lastly, we align two post-mortem sections from the striatum region of the human brain. One section contains spatial profiling of low molecular weight metabolites determined using (MALDI)-MSI mass-spectrometry, and the other section contains spatial profiling of mRNA transcripts measured using the 10x Genomics Visium technology (56). These sections are from a patient with Parkinson’s disease – a disorder characterized by dopamine depletion in the striatum (56). To evaluate MGW in comparison to other techniques, we assess the correspondence between the concentration of dopamine and its immediate breakdown products at various mass:charge ratios *m/z* in the spatial metabolomics dataset compared to the published annotations of Visium spots by dopamine-positive identity provided in (56) (Figure 6a). Specifically, (56) identifies 4 key dopamine metabolites, including DA Single (singly-derivatized dopamine) at *m/z* = 421.19, DA double (doubly-derivatized dopamine) at *m/z* = 674.28, the dopamine-breakdown product 3MT (3-Methoxytyramine) at *m/z* = 435.21, and the dopamine-breakdown product DOPAC double at *m/z* = 698.24. Dopamine-associated neurons were defined using the dopamine-high (Cd) annotations of the spatial transcriptomics slide (56).

We compared MGW to multi-modal single-cell alignment techniques moscot Translation (26), SCOT (11), and SCOTv2 (10), as well as two spatial-only baselines: PASTE2 with spatial information alone (30), and spatial-only GW. We choose the latter baselines in order to assess that the efficacy of the MGW alignment is not merely due to spatial information. Each method produces a coupling P between the locations on the metabolomics and spatial transcriptomics slices. We compute a barycentric projection (40) of the metabolite intensities through each P onto the ST coordinates. All methods except PASTE2, which performs partial alignment, transfer the full dopamine mass-intensity to the ST slide. We quantify alignment accuracy as as the overlap between projected dopamine intensities from the metabolomics slices and the annotated binary dopamine-high spot labels on the transcriptomics slice, and computed the AUROC and and AUPRC as a function of the predicted metabolite scores (Figure 6b). As the dopamine metabolites and dopamine-high spots form a weak, sharply localized signal (See Figure S8 for unscaled, raw-intensities), this benchmark assesses high-precision mapping of this small but biologically significant region. Low scores indicate failure to accurately map this local signal rather than poor global alignment.

MGW achieves the highest scores across all four metabolites (Table 1, Figure 6b). Specifically, the AUROC and AUPRC scores for 3MT, DA double, DA single, and DOPAC double were 0.991/0.846, 0.995/0.907, 0.994/0.882, and 0.951/0.455, respectively. These consistently exceeded the scores of other methods by large margins – for example, moscot Translation achieved 0.615/0.063, 0.590/0.058, 0.580/0.057, and 0.559/0.054, while the strongest spatial baseline PASTE2 reached 0.667/0.084, 0.674/0.090, 0.487/0.055, and 0.469/0.046. The alignment of Spatial GW performed near chance (AUROC ~ 0.52). Visualizations of the projections (Figure 6a) corroborate the observed quantitative trend: MGW correctly projects the dopamine metabolites onto the ground-truth dopamine-annotated regions with high spatial localization and accuracy. Other methods yield diffuse or incorrectly localized patterns, indicating that accurate spatial multiomic alignment requires both spatial and feature modalities. In this high-precision, localized prediction task, MGW is the only method which projects dopamine metabolites to the dopamine-annotated neurons.

## 4 Discussion

We introduce Manifold Gromov-Wasserstein (MGW) to align spatial multiomic data across heterogenous feature spaces. MGW assumes these spaces arise as the image of smooth maps from a shared Euclidean domain and learns modality-specific Riemannian metrics via neural fields. By using these Riemannian pull-back metrics, MGW compares the intrinsic structure of these modalities on their common spatial base, avoiding any hyperparameters balancing spatial and feature similarity. We demonstrate Manifold Gromov-Wasserstein on a diverse set of modalities – Visium and Stereo-seq sequencing, Xenium imaging, and MALDI-MSI and AFADESI-MSI mass-spectrometry metabolomics – highlighting the generality of MGW.

Several limitations suggest directions for future work. First, as MGW relies on two neural networks φ, ψ to learn the underlying cost for each modality space, the choice of underlying architecture may improve the quality of metric-learning and the alignment itself. While we focus on lightweight MLPs, a natural question is how the class of neural network may impact performance. Second, spatial multiomic data often contain modality-specific features with no shared structure. While we propose a pre-processing step based on CCA to mitigate this (Section C.6), we believe identifying mutually informative feature subspaces would improve estimation of the metric. Third, optimal transport variants such as unbalanced (46; 26; 10), semi-relaxed (17), and partial (30) OT often improve alignment robustness. Although MGW supports unbalanced marginals, we used balanced OT in our experiments to highlight the effect of the learned Manifold-GW cost.

Beyond spatial multiomic alignment, Riemannian distances may be especially advantageous for highly non-linear transformations such as spatiotemporal transcriptomics, where intrinsic manifold geometry is more informative than raw Euclidean distances (Section 3.1). Our ablations additionally indicate that the MGW Riemannian distances constitute a stronger GW term than either spatial or feature distances alone, suggesting FGW style frameworks may also benefit from adopting this cost.

Manifold Gromov-Wasserstein offers a principled way to align heterogeneous spatial omics datasets through deep Riemannian metric learning and optimal transport. More broadly, our results underscore that learning intrinsic geometry provides a powerful unifying principle for aligning nonlinear manifold data across the spatial multiomic universe.

## Supplementary Material

1

## Footnotes

1 The cost may depend on the coupling, as in (11).

2 Or, equivalently, a permutation $\sigma :(s^{i},X^{i})\to (t^{\sigma (i)},Z^{\sigma (i)})$

## A Appendix

### A.1 Pull-back Metrics and Base-Space Parametrization

Suppose we are given two Riemannian manifolds (ℳ,g) and (𝒩,h) and measures (datasets) μ∈𝒫(ℳ), ν∈𝒫(𝒩) supported on each space: for instance, for single-cell transcriptomics ℳ could represent an embedded sub-manifold of the d-dimensional measurements of transcript features $ℳ\subset R^{d}$, and for a distinct modality, e.g. metabolomics, 𝒩 could represent a sub-manifold of the p-dimensional measurements of metabolites $𝒩\subset R^{p}$ where we may have that p≠d and dim(ℳ)≠dim(𝒩). In general, it may appear to be a formidable task to align such datasets across such incomparable spaces, with no “natural” notion of distance immediately evident.

However, suppose that we instead take measurements on the product space ℳ×E and 𝒩×E for some common base manifold $\left(E,g^{E}\right)$. This may correspond to Euclidean space $E=R^{2}$ or $E=R^{3}$, e.g. when one reads the product of feature vectors with spatial coordinates as in spatial transcriptomics or spatial metabolomics. Recent, a remarkable line of work (6) have shown that one may learn an implicit neural representation of such product datasets. In particular, (6) shows that one may represent data (E,ℳ) as a smooth and differentiable map $\varphi \in C^{k}$ from a base-space E into a feature space ℳ,φ:E→ℳ. In other words, given spatial coordinates $s^{i}$ and product vector $p^{i}=(s^{i},X^{i})\in E\times ℳ$ one may instead represent $X^{i}≔\varphi (s^{i})$ and thus $p^{i}=(\mathrm{id},\varphi )\circ s^{i}$ for smooth, differentiable φ: a spatial transcriptomics dataset may be implicitly represented as a smooth function of its spatial coordinates. Thus, we state a first key assumption.

#### Assumption 1.

*Given* (ℳ,g) *and* (𝒩,h) *Riemannian, a common base manifold* $\left(E,g^{E}\right)$, *and points* $p^{i}=(s^{i},X^{i})\in E\times ℳ$ *and* $q^{j}=(s^{j},Z^{j})\in E\times 𝒩$ *we assume there there exist two* $C^{k}$ *local-parametrizations*

(13)
φ:E→ℳ,ψ:E→𝒩

*which satisfy*

$$X^{i}=p^{i}\;{|}_{ℳ}=\varphi (p^{i}\;{|}_{E})=\varphi (s^{i}),\;\;Z^{j}=q^{j}\;{|}_{𝒩}=\psi (q^{j}\;{|}_{E})=\psi (s^{j}).$$

As in (6), we optimize for such a pair of differentiable mappings with a neural network encoding each dataset implicitly. One solves this pair of maps φ, ψ as

$$\varphi ≔\varphi_{\theta },\;\;\theta =\underset{\theta }{\text{arg min}}\frac{1}{2n}\sum_{i=1}^{n}{\left\|\varphi_{\theta }(s^{i})-X^{i}\right\|}_{2}^{2},\;\psi ≔\psi_{\eta },\;\;\eta =\underset{\eta }{\text{arg min}}\frac{1}{2m}\sum_{j=1}^{m}{\left\|\psi_{\eta }(s^{j})-Z^{j}\right\|}_{2}^{2}$$

Given explicit maps φ, ψ from the base E into ℳ and 𝒩 with associated metrics on each space, there are natural vector fields defined through the mappings and a natural metric. In particular, for a vector field on E, V∈𝒳(E), one has associated push-forward vector fields on ℳ, 𝒩 given by

(14)
$$\varphi_{*}V(f)≔V(f\;\circ \;\varphi ),\;\;\psi_{*}V(g)≔V(g\;\circ \;\psi )$$

defined by ${\langle df,\varphi_{*}V\rangle }_{T_{\varphi (p)}ℳ}={\langle d(f\circ \varphi ),V\rangle }_{T_{p}E}$ for any test function $f\in C^{\infty }(ℳ)(\mathrm{resp}\%.𝒩)$. With a correspondence between spaces, we may measure manifold distances on each space with a *pull-back metric*.

#### Definition 3 (Pull-Back Metric.).

*Given manifolds* $\left(E,g^{E}\right)$, (𝒩,h) *and mapping* φ:E→𝒩, *the pull-back metric of* h *is the symmetric* (2, 0)*-tensor on* E *defined by*

(15)
$$\left(\psi^{*}h)(X,Y)=h(\psi_{*}(X),\psi_{*}(Y)\right)$$

From the duality of ${\langle df,\varphi_{*}V\rangle }_{T_{\varphi (p)}ℳ}={\langle d(f\circ \varphi ),V\rangle }_{T_{p}E}$, one identifies

$$h(\psi_{*}V,\psi_{*}V)=g^{E}(V,(d\psi^{T}\cdot h(\psi (p))\cdot d\psi )\;\circ \;V)$$

so that one may write this in terms of the Jacobian as

$${\left(\psi^{*}h\right)}_{\alpha ,\beta }\;(p)=h_{ij}(\psi (p))\frac{\partial \psi^{i}}{\partial x^{\alpha }}\frac{\partial \psi^{j}}{\partial x^{\beta }},\;x_{\alpha }x_{\beta }\;{\left(\psi^{*}h\right)}_{\alpha ,\beta }(p)={\left\|x\right\|}_{g_{p}^{M}}^{2},$$

For metric $g_{p}^{M}=D\varphi {\left(p\right)}^{T}h(\varphi (p))D\varphi (p):T_{p}E^{*}\times T_{p}E\to R_{+}$ defined at any point p∈E.

Now, suppose for simplicity that g and h are taken to be the standard Euclidean metrics $g_{\alpha \beta }=\delta_{\alpha \beta }$ in their respective spaces (i.e. in normal coordinates). Then, given a non-trivial pair of mappings φ, ψ one can find the following pull-back of both ℳ, 𝒩 to the common Euclidean space E:

$$\underset{\text{Manifold M Pull-Back}}{\underset{︸}{g^{M}(p)=D{\varphi (p)}^{T}D\varphi (p)}},\;\underset{\text{Manifold N Pull-Back}}{\underset{︸}{g^{N}(q)=D{\psi (p)}^{T}D\psi (q)}}$$

Thus, we arrive at a remarkable identification: if we measure in a product space E×ℳ and E×𝒩, and the pull-back maps ((φ,ψ), $g^{M}$) exist, one may pull manifold-distances back to a *common* base space. Observe that $g^{N}$ and E are *tensor fields* over p: each coordinate q, $g^{M}(p)$ has its own metric tensor $g^{N}(q)$ and E, which may vary signficantly over g.

### A.2 Metric Distances and Energy

Let us recall the fundamental notion of distance used with respect to a metric ds. The infinitesimal unit γ:[0,1]→E is known as the line element and gives an infinitesimal unit of arc length (distance). To extend this to distances over curves, define any curve over the manifold by t, and the line-element of this curve at any time γ(t) and point γ by

$$ds=\sqrt{g_{ij}(\gamma (t))(\dot{\gamma },\dot{\gamma })}dt$$

one measures the distance of a curve over time t with the arc-length integral

$$\int_{\gamma }ds=\int_{0}^{1}\sqrt{g_{ij}(\gamma (t))(\dot{\gamma },\dot{\gamma })}dt=L_{g}(\gamma )$$

For local coordinates $X=(x^{1},\cdots ,x^{n})$ one defines the infinitesimal squared-distance in g by

$$ds^{2}=g_{ij}(X)\;dx^{i}dx^{j},$$

So that the extension for a curve γ is $ds^{2}(\gamma )=g_{ij}(\gamma (t)){\dot{\gamma }}^{i}{\dot{\gamma }}^{j}$. The *energy* of a curve γ is defined as the integral of this squared-distance unit along the curve γ

$$E_{g}(\gamma )=\frac{1}{2}\int_{\gamma }ds^{2}=\frac{1}{2}\int_{0}^{1}g(\gamma )(\dot{\gamma },\dot{\gamma })\;dt≔\frac{1}{2}\int_{0}^{1}{\left\|\dot{\gamma }\right\|}_{g(\gamma )}^{2}dt$$

Suppose one normalizes γ to be parametrized by arc-length, i.e. the unique constant speed parametrization of γ so that ${\left\|\dot{\gamma }\right\|}_{2}=1$, then one may define a geodesic distance between any points $x^{a}$, $x^{b}$ by the minimal distance between these points over all curves γ connecting them:

$$d_{g}(x^{a},x^{b})=\underset{\gamma }{\text{inf}}\left\{L_{g}(\gamma )=\int_{0}^{1}\sqrt{g(\gamma (t))(\dot{\gamma },\dot{\gamma })}dt\;:\gamma \;:[0,1]\to E,\gamma (0)=x^{a},\gamma (1)=x^{b}\right\}$$

Which, for constant speed curves, is related to the energy by

$$d_{g}(x^{a},x^{b})=\underset{\gamma \;:x^{a}\to x^{b}}{\text{inf}}\;L_{g}(\gamma )=\underset{\gamma \;:x^{a}\to x^{b}}{\text{inf}}\sqrt{2E_{g}(\gamma )}.$$

The argmin of these problems is the unique constant-speed $\gamma^{*}$ from $x^{a}$ to $x^{b}$, and the minimum value of $d_{g}(x^{a},x^{b})$ is referred to as either a Riemannian distance or a geodesic distance between the pair of points. Thus, given a potentially point-dependent metric g on ℳ, one may define a geodesic distance between any pair of points. In the standard Euclidean case, the definitions above automatically recover the standard Euclidean distance and distance-squared, where $g_{ab}(x)=g_{ab}≔\delta_{ab}$ reduces to a trivial constant metric which lacks any dependence on the location of x on ℳ.

### A.3 Manifold Alignment via Geodesic Pull-Back Distances

Using the notions of distance above, we provide a formulation for the Gromov-Wasserstein problem (Definition 5) which offers a geometric unification of the alignment problem between spaces using the pull-back. In particular, this cost relies on geodesic pull-back distances under the pull-back metrics $g^{M}$, $g^{N}$ over the common base E.

#### Definition 4 (Riemannian Pull-Back Distances.).

*Suppose we are given two feature manifolds* (ℳ,g), (𝒩,h), *a common base manifold* $\left(E,g^{E}\right)$, *and points* $p^{i}=(s^{i},X^{i})\in E\times ℳ$ *and* $q^{j}=(s^{j},Z^{j})\in E\times 𝒩$. *Further, suppose that* Assumption 1 *holds with associated maps* φ:E→ℳ,ψ:E→𝒩. *We define the* M*-distance and* N*-distance of any two points* $x^{s},x^{t}\in E$ *to be the associated geodesic pull-back distances:*

dM(xs,xt)=infγ{LgM(γ)=∣∫01gM(γ(t))(γ.,γ.)dt∣γ:[0,1]→E,γ(0)=xs,γ(1)=xt},dN(xs,xt)resp.

*For pull-back metrics* $g^{M}$, $g^{N}$ *defined by*

$$d^{M}(p)=D{\varphi (p)}^{T}D\varphi (p),\;\;g^{N}(q)=D\psi {\left(q\right)}^{T}D\psi (q)$$

Now, given appropriate notions of pairwise distance in the common Euclidean space E, we may define the associated Gromov-Wasserstein (GW) problem (32; 33) as the natural notion of metric distortion between two spaces with two metrics.

#### Definition 5 (Manifold (Pull-back) Gromov-Wasserstein.).

*Given Riemannian pull-back distances* $d^{M}(\cdot ,\cdot )$ *and* $d^{N}(\cdot ,\cdot )$ (Definition 4*), define the associated squared distortions to be* $C^{M}(\cdot ,\cdot )≔d^{M}{\left(\cdot ,\cdot \right)}^{2}$, *and* $C^{N}(\cdot ,\cdot )≔d^{N}{\left(\cdot ,\cdot \right)}^{2}$. *For two distributions* $\mu^{M}\in 𝒫(E)$ *and* $\mu^{N}\in 𝒫(E)$, *the manifold pull-back GW Distance between* $\mu^{M}$ *and* $\mu^{N}$, *is*

(16)
$${\text{GW}}_{E}^{\left(M,N\right)}(\mu^{M},\mu^{N})=\underset{\pi \in \Pi (\mu^{M},\mu^{N})}{\text{min}}\int_{E\times E}{\left(C^{M}(x,x^{\prime })-C^{N}(z,z^{\prime })\right)}^{2}(d\pi ⊗d\pi )(x,z,x^{\prime },z^{\prime })$$

In other words, this is the standard Gromov-Wasserstein distance with Riemannian distance matrices built from Definition 4. Written explicitly in full, this is equal to

$$\underset{\pi \in \Pi (\mu^{M},\mu^{N})}{\text{min}}\int_{E\times E}{\left({\left(\underset{\gamma \in \Gamma (x,x^{\prime })}{\text{inf}}\int_{0}^{1}\sqrt{g^{M}(\dot{\gamma },\dot{\gamma })}dt\right)}^{2}-{\left(\underset{\gamma \in \Gamma (z,z^{\prime })}{\text{inf}}\int_{0}^{1}\sqrt{g^{N}(\dot{\gamma },\dot{\gamma })}dt\right)}^{2}\right)}^{2}(d\pi ⊗d\pi )(x,z,x^{\prime },z^{\prime })$$

The corresponding discrete problem for Definition 4 is given for two datasets of points (${\left(s^{i}\right)}_{i=1}^{n}\subset E$ and ${\left(t^{j}\right)}_{j=1}^{m}\subset E$ in the Euclidean base-space by

$$\underset{P\in \Pi (a,b)}{\text{min}}\sum_{ii^{\prime }}\sum_{jj^{\prime }}(C^{M}(s^{i},s^{i^{\prime }})-{C^{N}(t^{j},s^{j^{\prime }}))}^{2}P_{ij}P_{i^{\prime }j^{\prime }}\;≔\underset{P\in \Pi (a,b)}{\text{min}}\sum_{ii^{\prime }}\sum_{jj^{\prime }}{\left(C_{ii^{\prime }}^{M}-C_{jj^{\prime \prime }}^{N}\right)}^{2}P_{ij}P_{i^{\prime }j^{\prime }}$$

We summarize this discrete formulation in Problem 2. In the next section, we discuss a number of desirable properties and invariances of Definition 5 which makes it a natural option for aligning data that admits a product structure across two spaces.

## B Properties of the Gromov-Wasserstein Distance on the Metric Pull-back.

### B.1 Consistency under identity mapping

Observe that if φ,ψ≔id so that φ(s),ψ(t)≔s,t – i.e., there is no feature modality, one has that

$$g^{M}(p)=D{\varphi (p)}^{T}D\varphi (p)=1^{T}1=1,$$

$$g^{N}(q)=D{\psi (q)}^{T}D\psi (q)=1^{T}1=1$$

so that the metric tensor is the identity, and thus the geodesic distances become Euclidean distances, $d^{M}(x^{s},x^{t})={\left\|x^{s}-x^{t}\right\|}_{2}$. As a consequence, (16) simply reduces to a Gromov-Wasserstein problem on the raw spatial distances. Thus, (16) is consistent: in the case that no feature modality is present it recovers standard Gromov-Wasserstein on the spatial distances alone.

### B.2 Spatial Invariances

#### Proposition 1 (Spatial Invariances of Manifold Gromov-Wasserstein.).

Problem 2 *is invariant to arbitrary translations* $b\in R^{k}$, *and orthogonal transformations* $Q\in {𝒪}_{k}=\{Q\in R^{k\times k}:Q^{T}Q=QQ^{T}=I_{k}\}$, *so that solving 2 on* $s^{i}$ *is equivalent to solving on*

$${\tilde{s}}^{i}=Qs^{i}+b$$

*Proof.* Let $g^{N}=D\varphi^{T}D\varphi$ on a Euclidean manifold $E=R^{k}$. To assess spatial invariances, we consider which maps Φ:E→E applied to the points x→Φ(x) maintain the solution of the objective. Let us consider the action of the class of rigid-body transformations for arbitrary translations $b\in R^{k}$, and orthogonal transformation $Q\in R^{k\times k}:Q^{T}Q=QQ^{T}=I_{k}$. Suppose one learns the maps $\hat{\varphi }$, $\hat{\psi }$ over a rigid-body transformation of the coordinates y=Qx+b, $x=Q^{T}(y-b)$ and denote the maps over the original coordinates by φ, ψ. Since the features are identical under rotation of the spatial grid, one has that within the compact set Ω⊂E our points are supported on:

$$\hat{\varphi }(Qx+b)=\varphi (x),\;\;\hat{\psi }(Qx+b)=\psi (x)$$

and thus for the modified metric tensor ${\hat{g}}^{M}$ one identifies

$$D\hat{\varphi }(y)\;\circ \;Q=D\varphi (x),\;\;D\hat{\varphi }(y)=D\varphi (x)\;Q^{T}\;{\hat{g}}^{M}(y)=D\hat{\varphi }{\left(y\right)}^{T}D\hat{\varphi }(y)=Q\;D{\varphi (x)}^{T}D\varphi (x)\;Q^{T}=Q\;g^{M}(x)\;Q^{T}$$

implying that for any curve γ and its rigid transformation $\gamma_{*}=Q\gamma +b$ one has ${\dot{\gamma }}_{*}=Q\dot{\gamma }$ so that

$${\hat{g}}^{M}(y)({\dot{\gamma }}_{*},{\dot{\gamma }}_{*})={\hat{g}}^{M}(y)(Q\dot{\gamma },Q\dot{\gamma })\;\;={\left(Q\dot{\gamma }\right)}^{T}Qg^{M}(x)Q^{T}(Q\dot{\gamma })={\dot{\gamma }}^{T}Q^{T}Qg^{M}(x)Q^{T}Q\dot{\gamma }\;\;={\dot{\gamma }}^{T}g^{M}(x)\dot{\gamma }=g^{M}(x)(\dot{\gamma },\dot{\gamma })$$

So that infinitesimal line elements coincide, and

$${ds}_{{\hat{g}}^{M}}^{2}(\gamma_{*})={ds}_{g^{M}}^{2}(\gamma )$$

and integrating along curves yields that the geodesic distance in M (resp. N) is invariant under rigid motions

$$d_{\hat{g}}^{M}(Qu_{1}+b,Qu_{2}+b)=d_{g}^{M}(u_{1},u_{2})$$

### B.3 Feature Invariances

Similarly to the spatial case, for the feature case we evaluate which transformations $T^{\varphi }$, $T^{\psi }$ of the feature maps φ, ψ maintain the modified objective.

#### Proposition 2 (Feature Invariances of Manifold Gromov-Wasserstein.).

*Let* b, $b^{\prime }\in ℳ,𝒩$ *be any two constant vectors, let* λ∈R:λ≠0 *be a scaling, and* $Q\in {𝒪}_{d}$, $U\in {𝒪}_{p}$ *any two global orthogonal feature transformations. Then, the solution to* Problem 2 *is invariant to transformations of the feature space of the form*

$$\hat{\varphi }(x)=\lambda \cdot Q\varphi (x)+b,\;\;\hat{\psi }(x)=\lambda \cdot U\psi (x)+b^{\prime }$$

*Proof.* Let us focus on the following class of transformations for any constant vector b, $b^{\prime }\in ℳ$, 𝒩, a scaling λ∈R:λ≠0, and a global orthogonal feature transformation $Q\in {𝒪}_{d}$, $U\in {𝒪}_{p}$

$$\hat{\varphi }(x)=\lambda \cdot Q\varphi (x)+b,\;\;\hat{\psi }(x)=\lambda \cdot U\psi (x)+b^{\prime }$$

One may verify that the Jacobian is necessarily translation-invariant, yielding

$$D\hat{\varphi }(x)=\lambda QD\varphi (x),\;\;{\hat{g}}^{M}(x)={\left(\lambda QD\varphi (x)\right)}^{T}(\lambda QD\varphi (x))\;\;=\lambda^{2}D\varphi {\left(x\right)}^{T}Q^{T}QD\varphi (x)=\lambda^{2}D\varphi {\left(x\right)}^{T}D\varphi (x)=\lambda^{2}g^{M}(x)$$

With infinitesimal line-elements differing by a homogeneous constant, and implying

$$d_{\hat{g}}^{M}(u_{1},u_{2})=\lambda \cdot d_{g}^{M}(u_{1},u_{2})$$

So that, applying the same reasoning to $\hat{\psi }$, we have

$${\left(\lambda^{2}\cdot d_{g}^{M}(u_{1},u_{2}\right)}^{2}-{\lambda^{2}\cdot d_{g}^{N}{\left(u_{1}^{2},u_{2}^{\prime }\right)}^{2})}^{2}\;\;=\lambda^{4}{{\left(d_{g}^{M}(u_{1},u_{2}\right)}^{2}-d_{g}^{N}{\left(u_{1}^{\prime },u_{2}^{\prime }\right)}^{2})}^{2}$$

And have ${\mathrm{GW}}_{\hat{\varphi },\hat{\psi }}=\lambda^{4}\cdot {\mathrm{GW}}_{\varphi ,\psi }$, so that the solution is unchanged to this class of transformation and is conformally invariant to the change of metric $g\to \lambda^{2}g$.

**Table:**

Algorithm 1: Manifold GW (M-GW): Geodesic Distances via a pull-back metric on the common base E (Long Version)
$$\begin{array}{cc} \; & \;\mathrm{Input}\;:\text{Samples}\\ \; & \;\;\;\;\;\;\;{\left\{(s^{i},X^{i})\right\}}_{i=1}^{n}\subset E\times ℳ,\\ \; & \;\;\;\;\;\;\;{\left\{(t^{j},Z^{j})\right\}}_{j=1}^{m}\subset E\times 𝒩;\\ \; & \;\;\;\;\;\;\;\text{Base metric}\;g^{E}\;\text{on}\;E\;\text{e.g.},\text{Euclidean}).\\ \; & \;\mathrm{Output}:\text{Coupling}\;\pi^{⋆}\in \Pi (a,b)\;\text{aligning the two datasets}.\\ \; & \;(A)\;\mathrm{Learn}\;\mathrm{neural}\;\mathrm{maps}\;\mathrm{from}\;\mathrm{base}\;\mathrm{space}\;E\;\mathrm{to}\;\mathrm{feature}\;\mathrm{spaces}\\ \; & \;\;\;\;\;\text{Fit}\;\varphi_{\theta }\;\text{by}\;\theta \leftarrow {\text{arg min}}_{\theta }\frac{1}{n}\sum_{i}{\left\|\varphi_{\theta }(s^{i})-X^{i}\right\|}_{2}^{2};\\ \; & \;\;\;\;\;\text{Fit}\;\psi_{\eta }\;\text{by}\;\eta \leftarrow {\text{arg min}}_{\eta }\frac{1}{m}\sum_{i}{\left\|\psi_{\eta }(t^{j})-Z^{j}\right\|}_{2}^{2}.\\ \; & \;(B)\;\mathrm{Build}\;\mathrm{pull}\text{-}\mathrm{back}\;\mathrm{metrics}\;\mathrm{on}\;E\\ \; & \;\;\;\;\;\text{For each}\;s^{i}:\text{compute the Jacobian}\;J_{\varphi }(s^{i})=D\varphi (s^{i})\;(\text{via automatic differentiation})\;\text{and set}\\ \; & \;\;\;\;\;\;\;\;\;\;\;\;\;\;\;\;\;\;\;\;\;\;\;\;\;\;\;g^{M}(s^{i})≔J_{\varphi }{\left(s^{i}\right)}^{T}J_{\varphi }(s^{i}).\\ \; & \;\;\;\;\;\text{Foa each}\;t^{j}:\text{compute the Jacobian}\;J_{\psi }(t^{j})=D\psi (t^{j})\;\text{and set}\\ \; & \;\;\;\;\;\;\;\;\;\;\;\;\;\;\;\;\;\;\;\;\;\;\;\;\;\;\;g^{N}(t^{j})≔J_{\psi }{\left(t^{j}\right)}^{T}J_{\psi }(t^{j}).\\ \; & \;(C)\;\mathrm{Construct}\;\mathrm{base}\text{-}\mathrm{graphs}\;\mathrm{on}\;E\;\mathrm{with}\;\mathrm{local}\;\mathrm{arc}\text{-}\mathrm{lengths}.\\ \; & \;\;\;\;\;\text{On}\;\{s^{i}\}\;\text{build}\;k\text{-NN graph}\;{𝒢}_{M}.\;\text{For edge}\;(i,j)\;\text{define}\;\Delta_{ij}=(s^{i}-s^{j})\;\text{and compute weights}\\ \; & \;\;\;\;\;\;\;\;\;\;\;\;\;\;\;\;\;\;\;\;\;w_{M}^{ij}=\frac{1}{2}\sqrt{\Delta_{ij}^{T}g_{i}^{M}\Delta_{ij}}+\frac{1}{2}\sqrt{\Delta_{ij}^{T}g_{j}^{M}\Delta_{ij}}.\\ \; & \;\text{Analogously build}\;{𝒢}_{N}\;\text{on}\;\{t^{j}\}\;\text{with edge lengths}\;w_{N}^{jk}\;\text{from}\;g^{N}.\\ \; & \;(D)\;\mathrm{Geodesic}\;\mathrm{distance}\;\mathrm{on}\;E\\ \; & \;\;\;\;\;\text{Compute pairwise geodesic distances}\;d^{M}(s^{i},s^{i^{\prime }})\;\text{on}\;{𝒢}_{M};d^{N}(t^{j},t^{j^{\prime }})\;\text{on}\;{𝒢}_{N}.\\ \; & \;\text{Set}\;C_{ii^{\prime }}^{M}={\left(d^{M}(s^{i},s^{i^{\prime }})\right)}^{2},C_{jj^{\prime }}^{N}={\left(d^{N}(t^{j},t^{j^{\prime }}\right)}^{2}.\\ \; & \;(E)\;\mathrm{Solve}\;\mathrm{GW}\;\mathrm{with}\;\mathrm{pull}\text{-}\mathrm{back}\;\mathrm{geodesic}\;\mathrm{costs}\\ \; & \;\;\;\;\;\text{Given histograms}\;a\in \Delta^{n},b\in \Delta^{m},\;\text{solv}\\ \; & \;\;\;\;\;\;\;\;\;\;\;\;\;\;\;\;\;\;\;\;\pi^{⋆}\in \text{arg}\underset{\pi \in \Pi (a,b)}{\text{min}}\sum_{i,i^{\prime },j,j^{\prime }}{\left(C_{ii^{\prime }}^{M}-C_{jj^{\prime }}^{N}\right)}^{2}\pi_{ij}\pi_{i^{\prime }j^{\prime }}. \end{array}$$

## C Experimental Details

### C.1 Datasets, Availability, and Pre-processing.

In Section 3, we have evaluations of: (1) a spatiotemporal mouse transcriptomics Stereo-Seq dataset (5) (Section 3.1), (2) an evaluation of a Visium 10x Genomics colorectal cancer (CRC) dataset and a Xenium dataset at the same slide (37) (Section 3.2), (3) an alignment of a MALDI-MSI metabolomics dataset and Visium transcriptomics dataset of a human striatum brain section (56) (Section 3.4), and (4) and alignment of an AFADESI-MSI metabolomics and Visium transcriptomics dataset of human clear cell renal carcinoma (ccRCC) (53) (Section 3.3). All datasets are publicly accessible, and we publish the code for loading and processing each.

#### Stereo-Seq Spatiotemporal Transcriptomics.

For the dataset of (5) we use scanpy to load AnnData files for each day of mouse embryonic development from E9.5 to E13.5

- E9.5_E1S1.MOSTA.h5ad
- E10.5_E1S1.MOSTA.h5ad
- E11.5_E1S1.MOSTA.h5ad
- E12.5_E1S1.MOSTA.h5ad
- E13.5_E1S1.MOSTA.h5ad

We construct paired datasets by taking the intersection of common genes across the timepoints, and perform a joint PCA across the time pairs. To do this, we concatenate each consecutive timepoint pair and normalize the joint AnnData with sc.pp.normalize_total and add pseudo-counts with sc.pp.log1p for feature-sparsity. We then perform PCA on the joint AnnData with 30 components. As the PCA is performed jointly in this experiment, no additional CCA step is required for feature-alignment. Owing to the size of the slices, we randomly downsample each to 10k points with a random seed of rng=42.

#### Visium-Xenium Alignment.

For the public dataset of (37) we use the colorectal cancer (CRC) dataset. This consists of two multi-modal sections from the same donor (Sample P2 CRC) (37). This dataset includes a Visium CytAssist v2 section and a Xenium spatial transcriptomics section profiling the same tissue. We use this dataset, as it exhibits significant overlap (≈ 90%, (61)), making it an appropriate candidate for alignment. As the Visium technology offers sequencing of the transcriptomics (≈ 18, 000 genes), and Xenium offers FISH-based imagining (422 genes), we treat the datasets as distinct modalities. As a result, we perform independent PCA on each dataset (as opposed to a joint PCA) with 100 components. We then perform a CCA, as outlined in Section C.6, with 40 components to find the most mutually correlated components within the independent PCA sets.

#### Transcriptomics-Metabolomics of Human Striatum.

We download the Visium and MALDI-MSI datasets for the slide V11T17-102_A1 from (56), corresponding to a post-mortem slice of human striatum in with Parkinson’s diseases. The data deposition of (56) is available at Mendeley Data through the link https://data.mendeley.com/datasets/w7nw4km7xd/1. We select this slide in particular, as it is the only dataset with published annotations for (dopamine-annotated) Visium neurons which we use as a ground-truth in our benchmarking. These are contained in dopamine.csv in the directory V11T17-102/V11T17-102_A1/output_data/V11T17-102_A1_RNA/outs/dopamine.csv. We use the label dopamine_Cd as the ground-truth dopamine predictor in the caudate-nucleus (Cd), and use the labels not_dopamine_Cd and CI as the negative labels. We pre-process the metabolomics dataset with filtering for the top spatially-varying metabolites. We first normalize the metabolomics data with scanpy as sc.pp.normalize_total, and then use squidpy to compute the spatially variable metabolites with sq.gr.spatial_neighbors and sq.gr.spatial_autocorr, using mode="moran" to employ the Moran’s I statistic. We select the top 20 metabolites in this step, and use this pre-processed data for all methods. For MGW we do not perform any additional processing of the raw metabolomics data beyond filtering. We use the CCA step (Section C.6) to align the metabolomics and transcriptomics (PCA) features to 3 CC components in a joint-space suitable for MGW and use these features for alignment.

#### Transcriptomics-Metabolomics of Renal Cancer.

To benchmark against the multimodal model SpatialMeta, we employed the clear cell renal cell carcinoma (ccRCC) dataset from its original publication (53). The processed spatial transcriptomics (ST) and spatial metabolomics (SM) data for the Y7_T tissue section were obtained from the Zenodo repository (accession code 14986870), as Y7_T was the only slice for which both raw ST and SM data were publicly available. The corresponding ST-SM alignment generated by SpatialMeta was also downloaded from the same source.

For our method (MGW) and other optimal transport-based baselines, we followed SpatialMeta’s preprocessing workflow provided in their official GitHub repository (Vertical_and_Horizontal_Integration_for_ST_SM.ipynb ). To ensure a fair comparison, we adopted the same variational autoencoder (VAE) architecture as SpatialMeta across all methods and trained each model for 100 epochs to obtain the low-dimensional embeddings. We repeat the evaluation of the entire across three random seeds to ensure reproducibility.

Model evaluation was performed using SpatialMeta’s official benchmarking pipeline. In particular, this was done via the function run_modality_benchmark which is implemented in multi_benchmark_function.py in the benchmark/ folder. Since the manual pathological annotations referenced in the original publication were not included in the released dataset, metrics dependent on these annotations were excluded from our evaluation, and the remaining quantitative metrics were computed consistently across all models.

### C.2 Baselines

We benchmark MGW against the methods moscot (26), SCOT (11), SCOTv2 (10), SpatialMeta (53), and PASTE2 (30), in addition to two baselines: Gromov-Wasserstein optimal transport on (1) features and (2) spatial coordinates. The feature input is standardized for all methods to the PCA components computed by mgw.mgw_preprocess. We run moscot moscot.problems.TranslationProblem with max_iter=5_000 and tol=1e-7. Following the tutorial, available at https://moscot.readthedocs.io/en/latest/notebooks/tutorials/600_tutorial_translation.html, we use the default alpha = 1 as one must ignore the joint_attr for multiomics alignment. For SCOT we following the procedure outlined in the tutorial https://rsinghlab.github.io/SCOT/tutorial/. We set k=50 for the nearest neighbor graph, epsilon=0.005 for the entropic regularization, and set normalize=True. We use the same parameters for SCOTv2, with the additional parameter of rho=0.1. We run PASTE2 with s=0.7 and replace the adata.X coordinate with spatial coordinates, as multiomic alignment lacks joint distances across features. Lastly, for the Gromov-Wasserstein baselines, we use the package ott-jax (9) with feature and spatial costs constructed with squared-Euclidean distance as ${\left\|s^{i}-s^{j}\right\|}_{2}^{2}$ and ${\left\|t^{i}-t^{j}\right\|}_{2}^{2}$ (spatial), as well as ${\left\|X^{i}-X^{j}\right\|}_{2}^{2}$ and ${\left\|Z^{i}-Z^{j}\right\|}_{2}^{2}$ (feature in spaces ℳ and 𝒩). We use the parameters inner_maxit=3000, outer_maxit=3000, inner_tol=1e-8, outer_tol=1e-8, epsilon=1e-4 (Section 3.1), exactly paralleling the settings of ott-jax used for MGW but with a different cost. For the comparison to SpatialMeta, we downloaded their aligned adata_joint_Y7_T_raw.h5ad for Y7_T slice from their published Zenodo source https://zenodo.org/records/14986870. Then we trained the MGW, SpatialMeta and other models on ConditionalVAESTSM from SpatialMeta’s Github for 100 epochs to retrieve the embedding for downstream analysis.

### C.3 Architecture and Parameters.

To train our neural fields $\varphi :R^{2}\to ℳ$ and $\psi :R^{2}\to 𝒩$, we use multi-layer perceptrons (MLPs). For φ, ψ to be smooth sub-manifolds with sufficiently smooth Jacobians we require smooth $C^{\infty }$ activations: in particular, we exclude activation functions like ReLU and use the SiLU or SWISH function (41; 35). We use an MLP with widths (128, 256, 256, 128), learning rate $\eta =1\times 10^{-3}$, weight-decay of $w_{\text{decay}}=1\times 10^{-4}$, and EMA (exponential moving average) decay of ${\mathrm{EMA}}_{\text{decay}}=0.995$. We train the network for a total of niter=20_000 training iterations.

### C.4 Computing metric tensor-field and geodesics.

We use vmap(jacrev(phi))(X) to compute Jacobians at each point, and compute the pull-back metric tensor field using an Einstein summation of the form torch.einsum(‘nfd,nfe->nde’, J, J). We normalize this tensor-field across space with the mean-eigenvalue of g as a proxy, and add a small stabilization of the form $g_{\epsilon }^{M}\leftarrow g^{M}+\epsilon I$. As $g^{M}$ is scaled ≈ 1 we typically choose $\epsilon =10^{-2}$ to be much smaller, but sufficiently large to avoid instabilities for under-determined g.

To practically realize geodesics, we compute a K -nearest neighbor graph. On spatial positions $\left\{s^{i}\right\}$ we build k-NN graph ${𝒢}_{M}$. For edge (i,j) define $\Delta_{ij}=(s^{i}-s^{j})$. Then, the edge-weights in ${𝒢}^{M}$ are given by the arc-lengths in the Riemannian metric

$$w_{M}^{ij}=\frac{1}{2}\sqrt{\Delta_{ij}^{T}g_{i}^{M}\Delta_{ij}}+\frac{1}{2}\sqrt{\Delta_{ij}^{T}g_{j}^{M}\Delta_{ij}}$$

With $g^{M}$ defined from the neural field at each point $g^{M}(s^{i})(\cdot ,\cdot )$ and $g^{M}(s^{j})(\cdot ,\cdot )$. From this graph, geodesic distances are computed with Dykstra’s algorithm from scipy.sparse.csgraph.shortest_path.

### C.5 Details on the Optimal Transport.

For the optimal transport, we use the Gromov-Wasserstein solver of ott-jax (9). This relies on using our instantiated geodesic costs as

- geometry.Geometry(cost_matrix=CM)
- geometry.Geometry(cost_matrix=CN)

and calling quadratic_problem.QuadraticProblem on these objects. We use parameters of inner_maxit=3000, outer_maxit=3000, inner_tol=1e-8, outer_tol=1e-8, and epsilon=1e-4. We offer the unbalanced setting with τ_a and τ_b as options, but set both to 1 (Balanced) for all experiments.

### C.6 Pre-processing for the Alignment-Informative Feature Subset

The feature spaces of distinct modalities need not align nor exhibit meaningful joint structure. There may be “marginal” sub-spaces of each feature which may be independent of the other modality, and there may be “joint” sub-spaces which exhibit high-correlation across the modalities. Thus, a natural pre-processing is to filter for such joint sub-spaces.

We propose a step based on *canonical correlation analysis* (CCA) (20), which identifies linear combinations of two datasets which are maximally correlated. Formally, given random vectors $X\in R^{n}$, $Y\in R^{m}$ CCA computes a sequence of vectors ${\left(a_{k}\in R^{n}\right)}_{k=1}^{d}$, ${\left(b_{k}\in R^{m}\right)}_{k=1}^{p}$ that maximize the correlation of their projections $\rho =\mathrm{Corr}(a_{k}^{T}X,b_{k}^{T}Y)$. To isolate the joint sub-spaces, we propose an initial feature-refinement step comprising two components.

*First*, compute a coarse alignment $\hat{P}$ between the two datasets X and Z: an approximate correspondence is sufficient to identify features with strong cross-modal correspondence. *Second*, one takes a barycentric projection of the second dataset onto the first $Z^{\pi }=\hat{P}\;\mathrm{diag}(1∕{\hat{P}}^{T}1_{n})Z$. This ensures a 1-1 alignment of the points, which is a requirement of CCA. Following projection the canonical components are obtained by solving ${\text{arg max}}_{a_{k},b_{k}}\mathrm{Corr}(a_{k}^{T}X,b_{k}^{T}Z^{\pi })$ for k∈[d]. This yields canonically loading matrices $U_{a}$, $U_{b}$ with columns representing the maximally correlated directions of the two modalities. We then project the features in both modalities to their maximally-correlated components prior to MGW alignment. For a common modality, we perform a joint PCA across datasets in place of CCA.

## References

[1] Arvanitidis Georgios, González-Duque Miguel, Pouplin Alison, Kalatzis Dimitrios, and Hauberg Soren. Pulling back information geometry. In Camps-Valls Gustau, Ruiz Francisco J. R., and Valera Isabel, editors, Proceedings of The 25th International Conference on Artificial Intelligence and Statistics, volume 151 of Proceedings of Machine Learning Research, pages 4872–4894. PMLR, 28–30 Mar 2022.

[2] Baccin Chiara, Al-Sabah Jude, Velten Lars, Helbling Patrick M, Grünschläger Florian, Hernández-Malmierca Pablo, Nombela-Arrieta César, Steinmetz Lars M, Trumpp Andreas, and Haas Simon. Combined single-cell and spatial transcriptomics reveal the molecular, cellular and spatial bone marrow niche organization. Nature cell biology, 22(1):38–48, 2020. 31871321 10.1038/s41556-019-0439-6 PMC7610809

[3] Baydin Atılım Günes, Pearlmutter Barak A., Radul Alexey Andreyevich, and Siskind Jeffrey Mark. Automatic differentiation in machine learning: a survey. J. Mach. Learn. Res., 18(1):55955637, January 2017.

[4] Ben-Chetrit Nir, Niu Xiang, Swett Ariel D., Sotelo Jesus, Jiao Maria S., Stewart Caitlin M., Potenski Catherine, Mielinis Paulius, Roelli Patrick, Stoeckius Marlon, and Landau Dan A.. Integration of whole transcriptome spatial profiling with protein markers. Nature Biotechnology, 41(6):788793, January 2023. 10.1038/s41587-022-01536-3 PMC10272089 36593397

[5] Chen Ao, Liao Sha, Cheng Mengnan, Ma Kailong, Wu Liang, Lai Yiwei, Qiu Xiaojie, Yang Jin, Xu Jiangshan, Hao Shijie, Spatiotemporal transcriptomic atlas of mouse organogenesis using DNA nanoball-patterned arrays. Cell, 185(10):1777–1792, 2022. 35512705 10.1016/j.cell.2022.04.003

[6] Chitra Uthsav, Arnold Brian J., Sarkar Hirak, Sanno Kohei, Ma Cong, Lopez-Darwin Sereno, and Raphael Benjamin J.. Mapping the topography of spatial gene expression with interpretable deep learning. Nature Methods, 22(2):298309, January 2025. 10.1038/s41592-024-02503-3 PMC12370209 39849132

[7] Clifton Kalen, Anant Manjari, Aihara Gohta, Atta Lyla, Aimiuwu Osagie K, Kebschull Justus M, Miller Michael I, Tward Daniel, and Fan Jean. Stalign: Alignment of spatial transcriptomics data using diffeomorphic metric mapping. Nature communications, 14(1):8123, 2023. 10.1038/s41467-023-43915-7 PMC10709594 38065970

[8] Coifman R. R., Lafon S., Lee A. B., Maggioni M., Nadler B., Warner F., and Zucker S. W.. Geometric diffusions as a tool for harmonic analysis and structure definition of data: Diffusion maps. Proceedings of the National Academy of Sciences, 102(21):74267431, May 2005. 10.1073/pnas.0500334102 PMC1140422 15899970

[9] Cuturi Marco, Meng-Papaxanthos Laetitia, Tian Yingtao, Bunne Charlotte, Davis Geoff, and Teboul Olivier. Optimal Transport Tools (OTT): A JAX Toolbox for all things Wasserstein. arXiv preprint arXiv:2201.12324, 2022.

[10] Demetci Pinar, Santorella Rebecca, Chakravarthy Manav, Sandstede Bjorn, and Singh Ritambhara. Scotv2: Single-cell multiomic alignment with disproportionate cell-type representation. Journal of Computational Biology, 29(11):12131228, November 2022. 10.1089/cmb.2022.0270 PMC9805876 36251763

[11] Demetci Pinar, Santorella Rebecca, Sandstede Björn, Noble William Stafford, and Singh Ritambhara. Scot: single-cell multi-omics alignment with optimal transport. Journal of Computational Biology, 29(1):3–18, 2022. 35050714 10.1089/cmb.2021.0446 PMC8812493

[12] +Demetci Pinar, +Santorella Rebecca, +Sandstede Björn, +Noble William+Stafford, and +Singh Ritambhara. Gromov-wasserstein optimal transport to align single-cell multi-omics data. bioRxiv, 2020.

[13] Dong Kangning and Zhang Shihua. Deciphering spatial domains from spatially resolved transcriptomics with an adaptive graph attention auto-encoder. Nature Communications, 13(1), April 2022. 10.1038/s41467-022-29439-6 PMC8976049 35365632

[14] Fischer David S., Schaar Anna C., and Theis Fabian J.. Modeling intercellular communication in tissues using spatial graphs of cells. Nature Biotechnology, 41(3):332336, October 2022. 10.1038/s41587-022-01467-z PMC10017508 36302986

[15] Flamary RÃl’mi, Courty Nicolas, Gramfort Alexandre, Alaya Mokhtar Z., Boisbunon AurÃl’lie, Chambon Stanislas, Chapel Laetitia, Corenflos Adrien, Fatras Kilian, Fournier Nemo, Gautheron LÃl’o, Gayraud Nathalie T.H., Janati Hicham, Rakotomamonjy Alain, Redko Ievgen, Rolet Antoine, Schutz Antony, Seguy Vivien, Sutherland Danica J., Tavenard Romain, Tong Alexander, and Vayer Titouan. Pot: Python optimal transport. Journal of Machine Learning Research, 22(78):1–8, 2021.

[16] Halmos Peter, Gold Julian, Liu Xinhao, and Raphael Benjamin J.. Learning latent trajectories in developmental time series with Hidden-Markov optimal transport. In International Conference on Research in Computational Molecular Biology, pages 367–370. Springer, 2025.

[17] Halmos Peter, Liu Xinhao, Gold Julian, Chen Feng, Ding Li, and Raphael Benjamin J.. DeST-OT: Alignment of spatiotemporal transcriptomics data. Cell Systems, January 2025. 10.1016/j.cels.2024.12.001 PMC11972451 39874960

[18] Hao Yuhan, Hao Stephanie, Andersen-Nissen Erica, Mauck William M., Zheng Shiwei, Butler Andrew, Lee Maddie J., Wilk Aaron J., Darby Charlotte, Zager Michael, Hoffman Paul, Stoeckius Marlon, Papalexi Efthymia, Mimitou Eleni P., Jain Jaison, Srivastava Avi, Stuart Tim, Fleming Lamar M., Yeung Bertrand, Rogers Angela J., McElrath Juliana M., Blish Catherine A., Gottardo Raphael, Smibert Peter, and Satija Rahul. Integrated analysis of multimodal single-cell data. Cell, 184(13):3573–3587.e29, June 2021. 34062119 10.1016/j.cell.2021.04.048 PMC8238499

[19] Haviv Doron, Kunes Russell Zhang, Dougherty Thomas, Burdziak Cassandra, Nawy Tal, Gilbert Anna, and Pe’er Dana. Wasserstein wormhole: Scalable optimal transport distance with transformer. In Forty-first International Conference on Machine Learning, 2024.

[20] Hotelling H.. Relations between two sets of variates. Biometrika, 28(34):321377, December 1936.

[21] Hu Jian, Li Xiangjie, Coleman Kyle, Schroeder Amelia, Ma Nan, Irwin David J., Lee Edward B., Shinohara Russell T., and Li Mingyao. Spagcn: Integrating gene expression, spatial location and histology to identify spatial domains and spatially variable genes by graph convolutional network. Nature Methods, 18(11):13421351, October 2021. 10.1038/s41592-021-01255-8 34711970

[22] Hu Junyi, Wang Shao-Gang, Hou Yaxin, Chen Zhaohui, Liu Lilong, Li Ruizhi, Li Nisha, Zhou Lijie, Yang Yu, Wang Liping, Multi-omic profiling of clear cell renal cell carcinoma identifies metabolic reprogramming associated with disease progression. Nature Genetics, 56(3):442–457, 2024. 38361033 10.1038/s41588-024-01662-5 PMC10937392

[23] Huizing Geert-Jan, Samaran Jules, Capocefalo Daniele, Audit Anna, Peyré Gabriel, and Cantini Laura. Stories: learning cell fate landscapes from spatial transcriptomics using optimal transport. Nature Methods, November 2025. 10.1038/s41592-025-02855-4 PMC12982121 41184554

[24] Ji Andrew L, Rubin Adam J, Thrane Kim, Jiang Sizun, Reynolds David L, Meyers Robin M, Guo Margaret G, George Benson M, Mollbrink Annelie, Bergenstråhle Joseph, Multimodal analysis of composition and spatial architecture in human squamous cell carcinoma. Cell, 182(2):497–514, 2020. 32579974 10.1016/j.cell.2020.05.039 PMC7391009

[25] Kantorovich L. On the Translocation of Masses: Doklady akademii nauk ussr. 1942.

[26] Klein Dominik, Palla Giovanni, Lange Marius, Klein Michal, Piran Zoe, Gander Manuel, Meng-Papaxanthos Laetitia, Sterr Michael, Saber Lama, Jing Changying, Bastidas-Ponce Aimée, Cota Perla, Tarquis-Medina Marta, Parikh Shrey, Gold Ilan, Lickert Heiko, Bakhti Mostafa, Nitzan Mor, Cuturi Marco, and Theis Fabian J.. Mapping cells through time and space with moscot. Nature, January 2025. 10.1038/s41586-024-08453-2 PMC11864987 39843746

[27] Kühnel Wolfgang. Differential Geometry. American Mathematical Society, December 2015.

[28] Lee John M.. Introduction to Smooth Manifolds. Springer New York, 2012.

[29] Litinetskaya Anastasia, Schulman Maiia, Curion Fabiola, Szalata Artur, Omidi Alireza, Lotfollahi Mohammad, and Theis Fabian. Integration and Querying of Multimodal Single-Cell Data with PoE-VAE, page 345348. Springer Nature Switzerland, 2025.

[30] Liu Xinhao, Zeira Ron, and Raphael Benjamin J. Partial alignment of multislice spatially resolved transcriptomics data. Genome Research, 33(7):1124–1132, 2023. 37553263 10.1101/gr.277670.123 PMC10538490

[31] Ma Cong, Chitra Uthsav, Zhang Shirley, and Raphael Benjamin J.. Belayer: Modeling discrete and continuous spatial variation in gene expression from spatially resolved transcriptomics. Cell Systems, 13(10):786–797.e13, October 2022. 36265465 10.1016/j.cels.2022.09.002 PMC9814896

[32] Mémoli Facundo. On the use of Gromov-Hausdorff Distances for Shape Comparison. 2007.

[33] Mémoli Facundo. Gromov–Wasserstein Distances and the Metric Approach to Object Matching. Foundations of computational mathematics, 11:417–487, 2011.

[34] Miao Jishuai, Li Jinzhao, Xin Jingxue, Tu Jiajuan, Ge Muyang, Qi Ji, Zhou Xiaocheng, Zhu Ying, Yang Can, and Lin Zhixiang. Multigate: integrative analysis and regulatory inference in spatial multi-omics data via graph representation learning. Nature Communications, 16(1), October 2025. 10.1038/s41467-025-63418-x PMC12552752 41136370

[35] Misra Diganta. Mish: A self regularized non-monotonic activation function. Proceedings of the British Machine Vision Conference 2020, 2020.

[36] Moses Lambda and Pachter Lior. Museum of spatial transcriptomics. Nature Methods, 19(5):534–546, 2022. 35273392 10.1038/s41592-022-01409-2

[37] de Oliveira Michelli Faria, Romero Juan Pablo, Chung Meii, Williams Stephen R., Gottscho Andrew D., Gupta Anushka, Pilipauskas Susan E., Mohabbat Seayar, Raman Nandhini, Sukovich David J., Patterson David M., de Oliveira Michelli Faria, Romero Juan Pablo, Williams Stephen R., Patterson David M., Taylor Sarah E. B., and Taylor Sarah E. B.. High-definition spatial transcriptomic profiling of immune cell populations in colorectal cancer. Nature Genetics, 57(6):15121523, June 2025. 10.1038/s41588-025-02193-3 PMC12165841 40473992

[38] Palma Alessandro, Rybakov Sergei, Hetzel Leon, Günnemann Stephan, and Theis Fabian J. Enforcing latent euclidean geometry in single-cell VAEs for manifold interpolation. In Forty-second International Conference on Machine Learning, 2025.

[39] Paty François-Pierre and Cuturi Marco. Regularized optimal transport is ground cost adversarial. In Daumé Hal and Singh Aarti, editors, Proceedings of the 37th International Conference on Machine Learning, volume 119 of Proceedings of Machine Learning Research, pages 7532–7542. PMLR, 13–18 Jul 2020.

[40] Peyré Gabriel, Cuturi Marco, Computational optimal transport: With applications to data science. Foundations and Trends^®^ in Machine Learning, 11(5-6):355–607, 2019.

[41] Ramachandran Prajit, Zoph Barret, and Le Quoc V.. Searching for activation functions. CoRR, abs/1710.05941, 2017.

[42] Rao Anjali, Barkley Dalia, França Gustavo S, and Yanai Itai. Exploring tissue architecture using spatial transcriptomics. Nature, 596(7871):211–220, 2021. 34381231 10.1038/s41586-021-03634-9 PMC8475179

[43] Russell Andrew J. C., Weir Jackson A., Nadaf Naeem M., Shabet Matthew, Kumar Vipin, Kambhampati Sandeep, Raichur Ruth, Marrero Giovanni J., Liu Sophia, Balderrama Karol S., Vanderburg Charles R., Shanmugam Vignesh, Tian Luyi, Iorgulescu J. Bryan, Yoon Charles H., Wu Catherine J., Macosko Evan Z., and Chen Fei. Slide-tags enables single-nucleus barcoding for multimodal spatial genomics. Nature, 625(7993):101109, December 2023. 10.1038/s41586-023-06837-4 PMC10764288 38093010

[44] Saito Shinnosuke and Matsubara Takashi. Be tangential to manifold: Discovering riemannian metric for diffusion models, 2025.

[45] Sashittal Palash, Zhang Richard Y., Law Benjamin K., Strzalkowski Alexander, Schmidt Henri, Bolondi Adriano, Chan Michelle M., and Raphael Benjamin J.. Inferring cell differentiation maps from lineage tracing data. September 2024. 10.1038/s41592-025-02903-z PMC12982130 41360958

[46] Schiebinger Geoffrey, Shu Jian, Tabaka Marcin, Cleary Brian, Subramanian Vidya, Solomon Aryeh, Gould Joshua, Liu Siyan, Lin Stacie, Berube Peter, Optimal-Transport Analysis of Single-Cell Gene Expression Identifies Developmental Trajectories in Reprogramming. Cell, 176(4):928–943, 2019. 30712874 10.1016/j.cell.2019.01.006 PMC6402800

[47] Sha Yutong, Qiu Yuchi, Zhou Peijie, and Nie Qing. Reconstructing growth and dynamic trajectories from single-cell transcriptomics data. Nature Machine Intelligence, 6(1):2539, November 2023. 10.1038/s42256-023-00763-w PMC10805654 38274364

[48] Ståhl Patrik L, Salmén Fredrik, Vickovic Sanja, Lundmark Anna, Navarro José Fernández, Magnusson Jens, Giacomello Stefania, Asp Michaela, Westholm Jakub O, Huss Mikael, Visualization and analysis of gene expression in tissue sections by spatial transcriptomics. Science, 353(6294):78–82, 2016. 27365449 10.1126/science.aaf2403

[49] Stuart Tim, Butler Andrew, Hoffman Paul, Hafemeister Christoph, Papalexi Efthymia, Mauck William M., Hao Yuhan, Stoeckius Marlon, Smibert Peter, and Satija Rahul. Comprehensive integration of single-cell data. Cell, 177(7):1888–1902.e21, June 2019. 31178118 10.1016/j.cell.2019.05.031 PMC6687398

[50] Sun Xingzhi, Liao Danqi, MacDonald Kincaid, Zhang Yanlei, Huguet Guillaume, Wolf Guy, Adelstein Ian, Rudner Tim G. J., and Krishnaswamy Smita. Geometry-aware autoencoders for metric learning and generative modeling on data manifolds. In ICML 2024 Workshop on Geometry-grounded Representation Learning and Generative Modeling, 2024.

[51] Tanevski Jovan, Vulliard Loan, Ibarra-Arellano Miguel A., Schapiro Denis, Hartmann Felix J., and Saez-Rodriguez Julio. Learning tissue representation by identification of persistent local patterns in spatial omics data. Nature Communications, 16(1), April 2025. 10.1038/s41467-025-59448-0 PMC12044154 40307222

[52] Tenenbaum Joshua B., de Silva Vin, and Langford John C.. A global geometric framework for nonlinear dimensionality reduction. Science, 290(5500):23192323, December 2000. 10.1126/science.290.5500.2319 11125149

[53] Tian Ruonan, Xue Ziwei, Chen Yiru, Qi Yicheng, Zhang Jian, Yuan Jie, Ruan Dengfeng, Lin Junxin, Liu Jia, Wang Di, Ye Youqiong, and Liu Wanlu. Integrating cross-sample and cross-modal data for spatial transcriptomics and metabolomics with spatialmeta. Nature Communications, 16(1), October 2025. 10.1038/s41467-025-63915-z PMC12500918 41053019

[54] Titouan Vayer, Courty Nicolas, Tavenard Romain, and Flamary Rémi. Optimal transport for structured data with application on graphs. In International Conference on Machine Learning, pages 6275–6284. PMLR, 2019.

[55] Vayer Titouan, Chapel Laetitia, Flamary Remi, Tavenard Romain, and Courty Nicolas. Fused Gromov-Wasserstein distance for structured objects. Algorithms, 13(9):212, August 2020.

[56] Vicari Marco, Mirzazadeh Reza, Nilsson Anna, Shariatgorji Reza, Bjärterot Patrik, Larsson Ludvig, Lee Hower, Nilsson Mats, Foyer Julia, Ekvall Markus, Czarnewski Paulo, Zhang Xiaoqun, Svenningsson Per, Käll Lukas, Andrén Per E., and Lundeberg Joakim. Spatial multimodal analysis of transcriptomes and metabolomes in tissues. Nature Biotechnology, 42(7):10461050, September 2023. 10.1038/s41587-023-01937-y PMC11251988 37667091

[57] Welch Joshua D., Kozareva Velina, Ferreira Ashley, Vanderburg Charles, Martin Carly, and Macosko Evan Z.. Single-cell multi-omic integration compares and contrasts features of brain cell identity. Cell, 177(7):1873–1887.e17, June 2019. 31178122 10.1016/j.cell.2019.05.006 PMC6716797

[58] Xu Chang, Jin Xiyun, Wei Songren, Wang Pingping, Luo Meng, Xu Zhaochun, Yang Wenyi, Cai Yideng, Xiao Lixing, Lin Xiaoyu, Liu Hongxin, Cheng Rui, Pang Fenglan, Chen Rui, Su Xi, Hu Ying, Wang Guohua, and Jiang Qinghua. Deepst: identifying spatial domains in spatial transcriptomics by deep learning. Nucleic Acids Research, 50(22):e131e131, October 2022. 36250636 10.1093/nar/gkac901 PMC9825193

[59] Zeira Ron, Land Max, Strzalkowski Alexander, and Raphael Benjamin J. Alignment and integration of spatial transcriptomics data. Nature Methods, 19(5):567–575, 2022. 35577957 10.1038/s41592-022-01459-6 PMC9334025

[60] Zhang Di, Deng Yanxiang, Kukanja Petra, Agirre Eneritz, Bartosovic Marek, Dong Mingze, Ma Cong, Ma Sai, Su Graham, Bao Shuozhen, Liu Yang, Xiao Yang, Rosoklija Gorazd B., Dwork Andrew J., Mann J. John, Leong Kam W., Boldrini Maura, Wang Liya, Haeussler Maximilian, Raphael Benjamin J., Kluger Yuval, Castelo-Branco Gonçalo, and Fan Rong. Spatial epigenometranscriptome co-profiling of mammalian tissues. Nature, 616(7955):113122, March 2023. 10.1038/s41586-023-05795-1 PMC10076218 36922587

[61] Zheng Hongyu, Sarkar Hirak, and Raphael Benjamin J.. Joint imputation and deconvolution of gene expression across spatial transcriptomics platforms. February 2025. 10.1101/gr.280555.125 PMC12667712 41249066

