# Supplementary material for: Riemannian Metric Learning for Alignment of Spatial Multiomics
Source: bioRxiv. 2025 Dec 11:2025.12.09.693237. Preprint. [Version 1] doi: 10.64898/2025.12.09.693237 (PMC12713592; doi:10.64898/2025.12.09.693237)
Supplement: 1 [file NIHPP2025.12.09.693237v1-supplement-1.pdf]

bioRxiv preprint doi: https://doi.org/10.64898/2025.12.09.693237; this version posted December 11, 2025. The copyright holder for this preprint (which was not certified by peer review) is the author/funder, who has granted bioRxiv a license to display the preprint in perpetuity. It is made available under aCC-BY 4.0 International license.

D Supplementary Figures and Tables

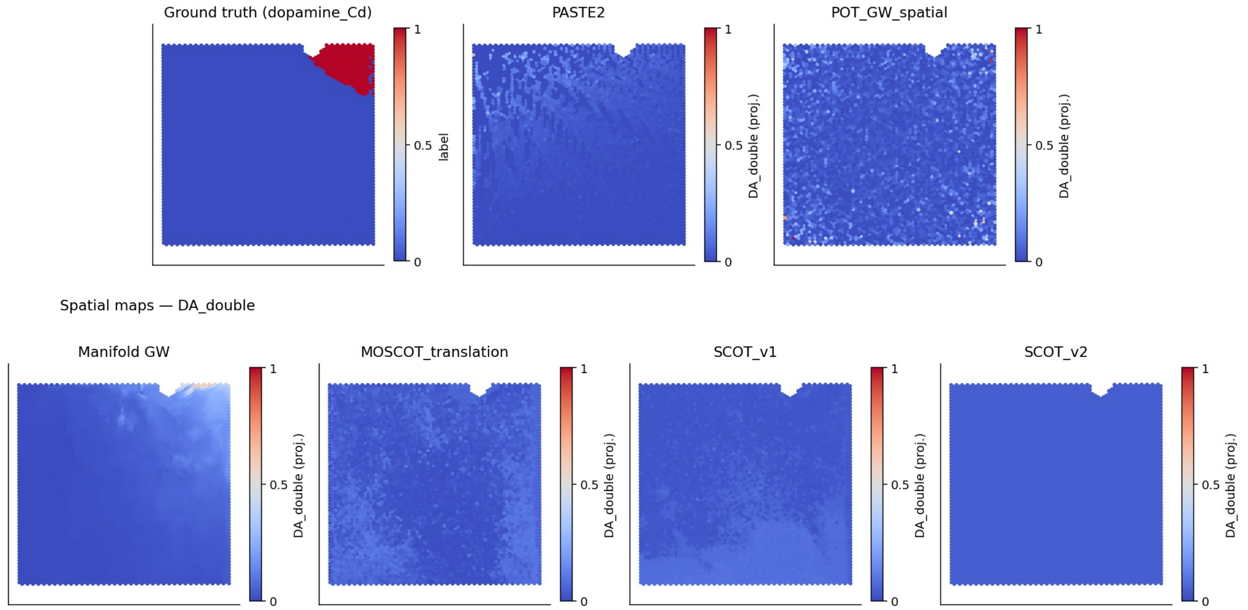

Figure S7: Raw, unscaled DA Double (doubly-derivatized dopamine, $m/z = 674.28$) following barycentric projection of metabolite intensities onto Visium Slide of (56) across couplings $\boldsymbol{P}$ returned by various methods.

bioRxiv preprint doi: https://doi.org/10.64898/2025.12.09.693237; this version posted December 11, 2025. The copyright holder for this preprint (which was not certified by peer review) is the author/funder, who has granted bioRxiv a license to display the preprint in perpetuity. It is made available under aCC-BY 4.0 International license.

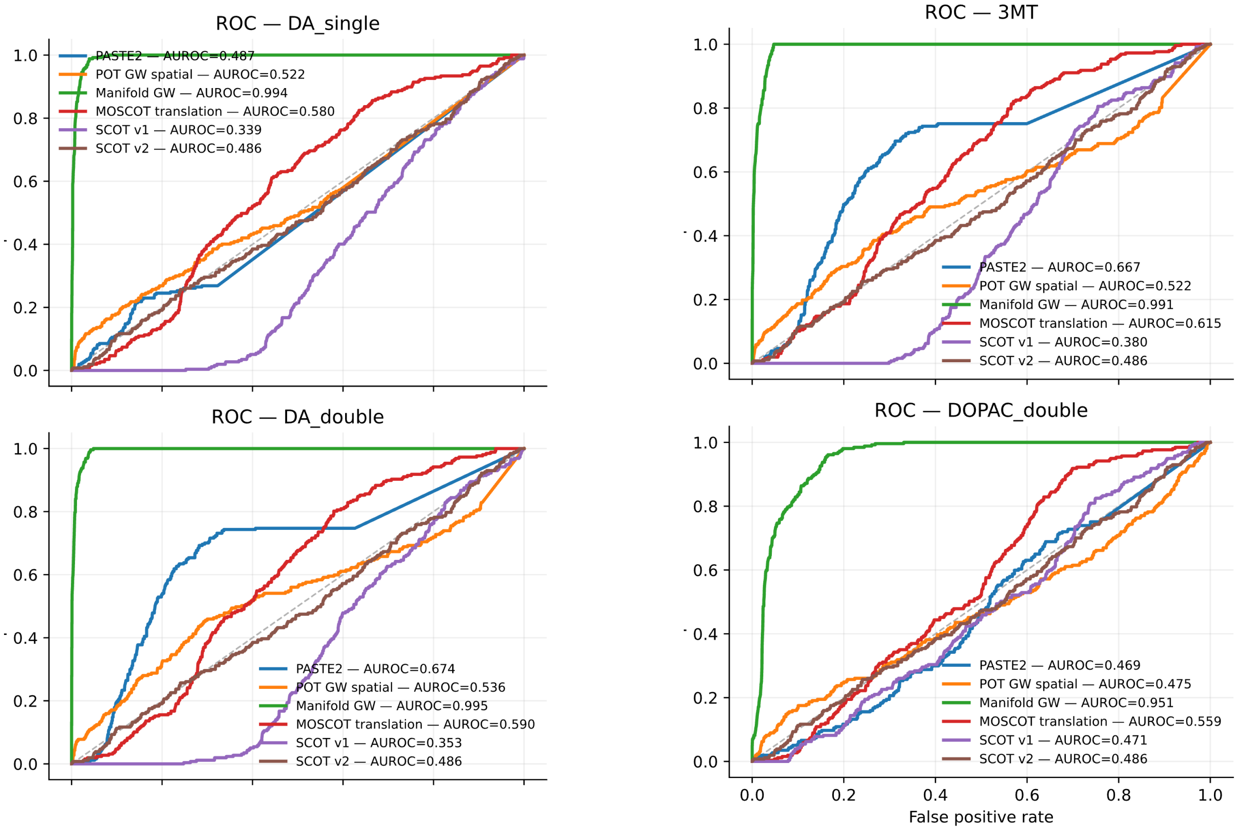

Figure S8: AUROC curves for the 4 dopamine metabolites DA Single (singly-derivatized $m/z = 421.19$), DA double (doubly-derivatized $m/z = 674.28$), DOPAC double (dopamine-breakdown product $m/z = 698.24$), and 3MT (dopamine-breakdown product 3-Methoxytyramine, $m/z = 435.21$) in the MALDI-MSI to Visium Transfer Task.

bioRxiv preprint doi: https://doi.org/10.64898/2025.12.09.693237; this version posted December 11, 2025. The copyright holder for this preprint (which was not certified by peer review) is the author/funder, who has granted bioRxiv a license to display the preprint in perpetuity. It is made available under aCC-BY 4.0 International license.

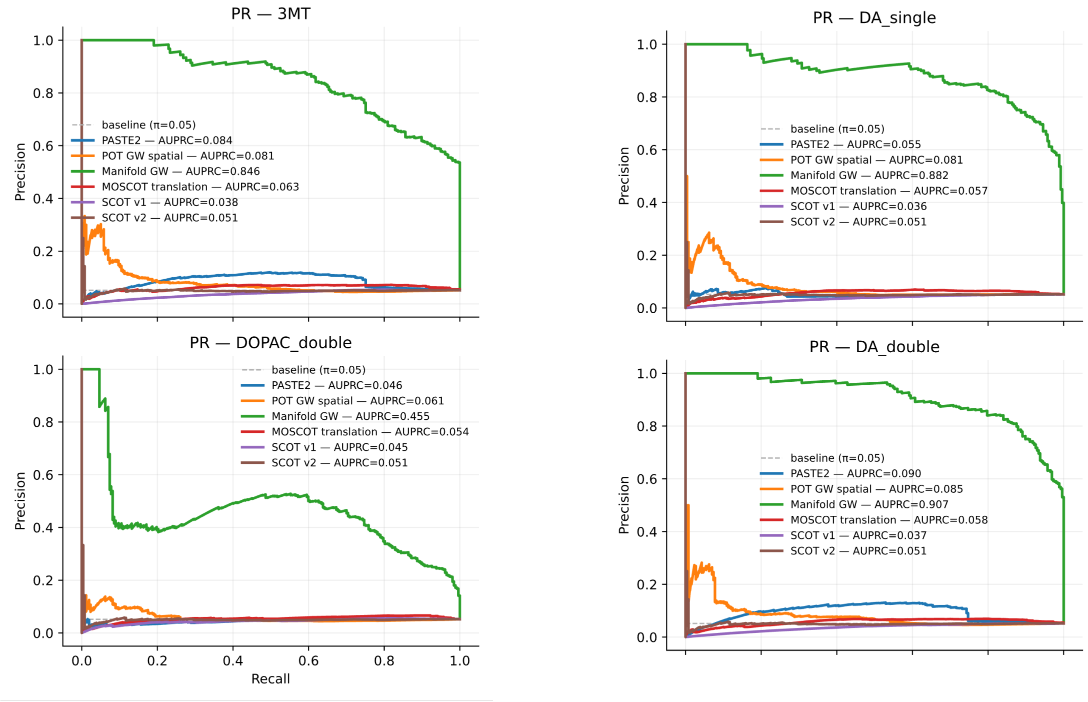

Figure S9: AUPRC curves for the 4 dopamine metabolites DA Single (singly-derivatized $m/z = 421.19$), DA double (doubly-derivatized $m/z = 674.28$), DOPAC double (dopamine-breakdown product $m/z = 698.24$), and 3MT (dopamine-breakdown product 3-Methoxytyramine, $m/z = 435.21$) in the MALDI-MSI to Visium Transfer Task.

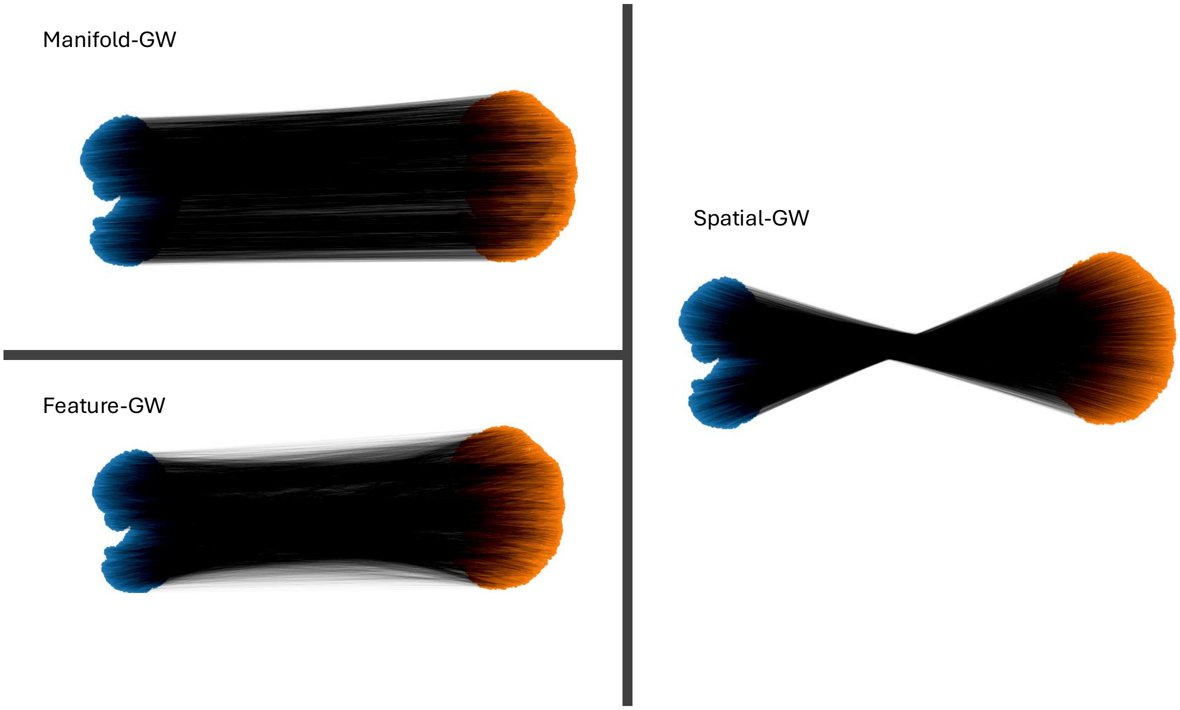

Figure S10: Visualization of alignments computed by Manifold-GW, as well as Gromov-Wasserstein baselines Spatial-only GW and Feature-only GW. Alignments shown for Stereo-seq mouse-embryo timepairs E12.5-13.5 from (5).

bioRxiv preprint doi: https://doi.org/10.64898/2025.12.09.693237; this version posted December 11, 2025. The copyright holder for this preprint (which was not certified by peer review) is the author/funder, who has granted bioRxiv a license to display the preprint in perpetuity. It is made available under aCC-BY 4.0 International license.

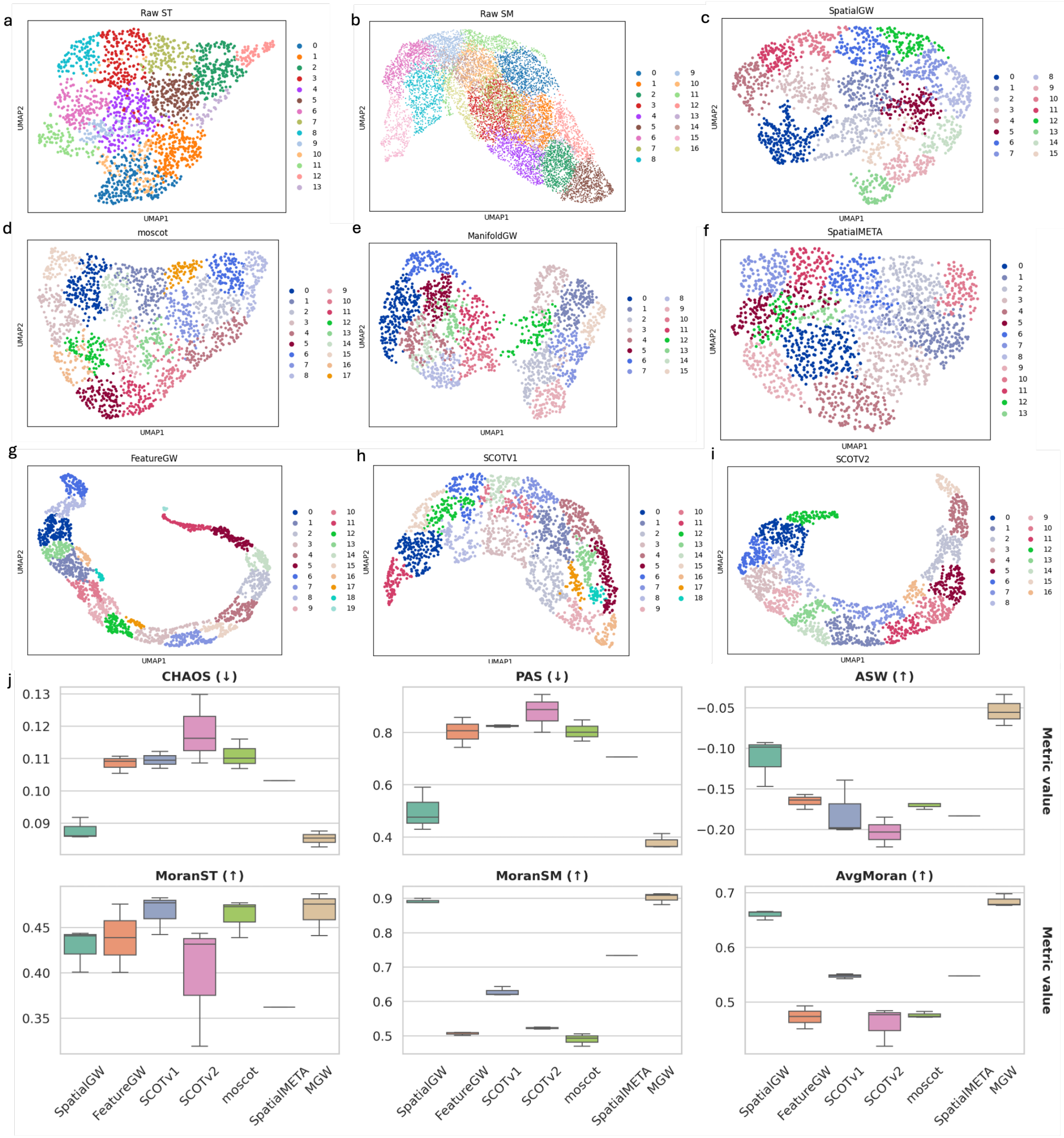

Figure S11: **(a-i)** The UMAP visualization of the aligned embeddings of Y_T slice from ccRCC dataset of all models.**(j)** The boxplot of all six metrics across three seeds as in table 2. The SpatialMeta's value is consistent across three seeds, because it is not an optimal transport based model and there is no randomness coming from the approximation of coupling (53). The up or down arrow next to the metric stands for if a higher or lower value means better performance.

bioRxiv preprint doi: https://doi.org/10.64898/2025.12.09.693237; this version posted December 11, 2025. The copyright holder for this preprint (which was not certified by peer review) is the author/funder, who has granted bioRxiv a license to display the preprint in perpetuity. It is made available under aCC-BY 4.0 International license.

Table 1: Comparison of alignment methods across dopamine-related MSI targets. Best scores per target are shown in bold.

**Method**	**Target**	m/z	**AUROC**	**AUPRC**
Manifold GW	3MT	435.21	**0.991**	**0.846**
PASTE2	3MT	435.21	0.667	0.084
MOSCOT (translation)	3MT	435.21	0.615	0.063
POT–GW (spatial)	3MT	435.21	0.522	0.081
SCOT_v2	3MT	435.21	0.486	0.051
SCOT_v1	3MT	435.21	0.380	0.038
Manifold GW	DA_double	674.28	**0.995**	**0.907**
PASTE2	DA_double	674.28	0.674	0.090
MOSCOT (translation)	DA_double	674.28	0.590	0.058
POT–GW (spatial)	DA_double	674.28	0.536	0.085
SCOT_v2	DA_double	674.28	0.486	0.051
SCOT_v1	DA_double	674.28	0.353	0.037
Manifold GW	DA_single	421.19	**0.994**	**0.882**
MOSCOT (translation)	DA_single	421.19	0.580	0.057
POT–GW (spatial)	DA_single	421.19	0.522	0.081
PASTE2	DA_single	421.19	0.487	0.055
SCOT_v2	DA_single	421.19	0.486	0.051
SCOT_v1	DA_single	421.19	0.339	0.036
Manifold GW	DOPAC_double	698.24	**0.951**	**0.455**
MOSCOT (translation)	DOPAC_double	698.24	0.559	0.054
SCOT_v2	DOPAC_double	698.24	0.486	0.051
POT–GW (spatial)	DOPAC_double	698.24	0.475	0.061
SCOT_v1	DOPAC_double	698.24	0.471	0.045
PASTE2	DOPAC_double	698.24	0.469	0.046

bioRxiv preprint doi: https://doi.org/10.64898/2025.12.09.693237; this version posted December 11, 2025. The copyright holder for this preprint (which was not certified by peer review) is the author/funder, who has granted bioRxiv a license to display the preprint in perpetuity. It is made available under aCC-BY 4.0 International license.

Table 2: Comparison of alignment methods across metrics across three random seeds. Best scores per metric are shown in bold.

Method	CHAOS	PAS	ASW	Moran's I (ST)	Moran's I (SM)	Avg Moran's I
SpatialGW	0.0918	0.5906	-0.0984	0.4007	0.8998	0.6502
FeatureGW	0.1092	0.8582	-0.1568	0.4389	0.5091	0.4740
SCOTv1	0.1095	0.8290	-0.2004	0.4771	0.6193	0.5482
SCOTv2	0.1298	0.9459	-0.2033	0.3194	0.5198	0.4196
moscot	0.1101	0.8012	-0.1682	0.4389	0.5061	0.4725
SpatialMETA	0.1032	0.7063	-0.1834	0.3623	0.7334	0.5478
MGW	**0.0876**	**0.4127**	**-0.0717**	**0.4869**	**0.9090**	**0.6979**
SpatialGW	0.0858	0.4757	-0.1469	0.4406	0.8880	0.6643
FeatureGW	0.1054	0.7433	-0.1636	0.4006	0.5017	0.4511
SCOTv1	0.1070	0.8211	-0.1391	0.4421	0.6435	0.5428
SCOTv2	0.1086	0.8012	-0.1849	0.4436	0.5255	0.4845
moscot	0.1069	0.7675	-0.1752	**0.4728**	0.4931	0.4829
SpatialMETA	0.1032	0.7063	-0.1834	0.3623	0.7334	0.5478
MGW	**0.0854**	**0.3642**	**-0.0556**	0.4410	**0.9130**	**0.6770**
SpatialGW	0.0861	0.4291	-0.0928	0.4436	**0.8878**	0.6657
FeatureGW	0.1107	0.8072	-0.1752	0.4758	0.5102	0.4930
SCOTv1	0.1122	0.8265	-0.1978	**0.4826**	0.6209	0.5517
SCOTv2	0.1163	0.8880	-0.2214	0.4315	0.5224	0.4769
moscot	0.1160	0.8488	-0.1681	0.4771	0.4701	0.4736
SpatialMETA	0.1032	0.7063	-0.1834	0.3623	0.7334	0.5478
MGW	**0.0827**	**0.3622**	**-0.0336**	0.4758	0.8815	**0.6786**
**Average**						
SpatialGW	0.0879	0.4985	-0.1127	0.4283	0.8919	0.6601
FeatureGW	0.1084	0.8029	-0.1652	0.4384	0.5070	0.4727
SCOTv1	0.1096	0.8255	-0.1791	0.4673	0.6279	0.5476
SCOTv2	0.1182	0.8784	-0.2032	0.3982	0.5226	0.4603
moscot	0.1110	0.8058	-0.1705	0.4629	0.4898	0.4763
SpatialMETA	0.1032	0.7063	-0.1834	0.3623	0.7334	0.5478
MGW	**0.0852**	**0.3797**	**-0.0536**	**0.4679**	**0.9012**	**0.6845**

bioRxiv preprint doi: https://doi.org/10.64898/2025.12.09.693237; this version posted December 11, 2025. The copyright holder for this preprint (which was not certified by peer review) is the author/funder, who has granted bioRxiv a license to display the preprint in perpetuity. It is made available under aCC-BY 4.0 International license.

Table 3: Pairwise alignment performance across developmental stages. Lower migration and higher projected AMI indicate better cross-slice alignment quality.

Pair	Method	Migration ↓	Proj. AMI (A↔B) ↑
E9.5→E10.5	FeatureGW	0.473	0.455
E9.5→E10.5	MGW	0.077	0.353
E9.5→E10.5	MOSCOT_translation	0.508	0.389
E9.5→E10.5	SCOT_v2	0.510	0.055
E9.5→E10.5	SpatialGW	0.050	0.315
E10.5→E11.5	FeatureGW	0.450	0.352
E10.5→E11.5	MGW	0.120	0.393
E10.5→E11.5	MOSCOT_translation	0.465	0.420
E10.5→E11.5	SCOT_v2	0.499	0.195
E10.5→E11.5	SpatialGW	0.044	0.345
E11.5→E12.5	FeatureGW	0.489	0.304
E11.5→E12.5	MGW	0.172	0.364
E11.5→E12.5	MOSCOT_translation	0.469	0.400
E11.5→E12.5	SCOT_v2	0.491	0.163
E11.5→E12.5	SpatialGW	0.463	0.341
E12.5→E13.5	FeatureGW	0.492	0.319
E12.5→E13.5	MGW	0.112	0.338
E12.5→E13.5	MOSCOT_translation	0.478	0.368
E12.5→E13.5	SCOT_v2	0.498	0.080
E12.5→E13.5	SpatialGW	0.478	0.236

Table 4: Comparison of alignment methods on cosine similarity and migration metrics for Visium-Xenium alignment (CRC Colorectal Cancer tumor sample).

Method	Mean Cosine	Median Cosine	Migration
MGW	0.671	0.689	0.014
Spatial GW	0.660	0.681	0.218
Feature GW	0.744	0.753	0.239
Moscot	0.742	0.754	0.234
SCOT	0.665	0.694	0.234
SCOTv2	0.714	0.725	0.248
PASTE	0.437	0.455	0.001
POT	0.722	0.738	0.278
